# Synthesis and crystal structures of bis­[1-oxopyridin-2-olato(1−)]bis­(penta­fluoro­phen­yl)silicon(IV)–tetra­hydro­furan–pentane (2/1/1), bis­[1-oxopyridin-2-olato(1−)]bis­(*p*-tol­yl)silicon(IV), and dimesitylbis[1-oxopyridin-2-olato(1−)]silicon(IV)

**DOI:** 10.1107/S2056989024001543

**Published:** 2024-02-20

**Authors:** Bradley M. Kraft, William W. Brennessel, Jordan W. Andrews, Michael T. Viggiani, Nathan F. Kittrell, Matthew T. Heckman

**Affiliations:** a Department of Chemistry, St. John Fisher University, Rochester, NY 14618, USA; b Department of Chemistry, 120 Trustee Road, University of Rochester, Rochester, NY 14627, USA; Universidade Federal do ABC, Brazil

**Keywords:** crystal structure, silicon, pyridinone, pyridine *N*-oxide

## Abstract

The neutral organosilicon(IV) complex, (C_6_F_5_)_2_Si(OPO)_2_ (OPO = 1-oxopyridin-2-one, C_5_H_4_NO_2_), was synthesized from (C_6_F_5_)_2_Si(OCH_3_)_2_ and 2 equiv. of 1-hy­droxy­pyridin-2-one in tetra­hydro­furan (THF). Single crystals grown from the diffusion of *n*-pentane into a THF solution were identified as a THF hemisolvate and an *n*-pentane hemisolvate, (C_6_F_5_)_2_Si(OPO)_2_·0.5THF·0.5C_5_H_12_ (**1**). *p*-Tol­yl_2_Si(OPO)_2_ (**2**) and mesit­yl_2_Si(OPO)_2_ (**3**) crystallized directly from reaction mixtures of 2 equiv. of Me_3_Si(OPO) with *p*-tol­yl_2_SiCl_2_ and mesit­yl_2_SiCl_2_, respectively, in aceto­nitrile. The oxygen-bonded carbon and nitro­gen atoms of the OPO ligands in **1**, **2**, and **3** were modeled as disordered indicating co-crystallization of up to three possible diastereomers in each. Solution NMR studies support the presence of exclusively the all-*cis* isomer in **1** and multiple isomers in **2**. Poor solubility of **3** limited its characterization in solution.

## Abstract 2

Three hexa­coordinated bis­(ar­yl)silicon(IV) complexes of 1-oxopyridin-2-one (OPO) are reported, each of which exhibit C/N site disorder in their pyridine rings. In (C_6_F_5_)_2_Si(OPO)_2_, the equal disorder ratios and solution NMR characterization together indicate the presence of a single totally asymmetric ON-*trans*-OC isomer. Unequal disorder ratios in *p*-tol­yl_2_Si(OPO)_2_ and in mesit­yl_2_Si(OPO)_2_ indicate the presence of up to three isomers.

## 1. Chemical context

The intriguing capacity of 1-hy­droxy­pyridin-2-one (HOPO) to dissolve silica to form [Si(OPO)_3_]^+^ in aqueous solution was reported by Weiss & Harvey in 1964 ▸. More recently, related ligand derivatives have been utilized as sequestering agents of lead and rare-earth metals, among others (Lewis & Cohen, 2004 ▸; Szigethy & Raymond, 2011 ▸; Wang, *et al.*, 2019 ▸). In order to further study the powerful chelate effect of the OPO ligand, we have examined the solution- and solid-state structures of silicon complexes with varying organo ancillary ligands.

Previously reported hexa­coordinate neutral di­alkyl­silicon 1-oxopyridin-2-one (OPO) complexes, *R*_2_Si(OPO)_2_ [*R* = Me, Et, *i*Pr; *R*_2_ = (CH_2_)_3_], and one diaryl complex, Ph_2_Si(OPO)_2_, each exhibit co-crystallization of up to three possible isomers due, in part, to the isosteric character of the OPO ligand with the coplanar flip of itself (Kraft & Brennessel, 2014 ▸). In solution at room temperature, the dialkyl complexes exhibit only five OPO ligand resonances by NMR spectroscopy, indicating rapid inter­conversion of isomers that occurs with concomitant Si←OC bond dissociation. For Me_2_Si(OPO)_2_, three isomers were observed at 193 K by ^1^H NMR spectros­copy. In Ph_2_Si(OPO)_2_, the more electron-withdrawing phenyl groups strengthened the OPO ligand chelate inter­action as given by generally shorter Si—O distances, and this resulted also in a slower inter­conversion between isomers relative to the alkyl derivatives (Kraft & Brennessel, 2014 ▸).

In all known *R*_2_Si(OPO)_2_ complexes, the pair of Si—O bond distances *trans* to alkyl or aryl groups are longer than those *cis*. This characteristic, together with the observed C/N site disorder, highlights the underlying ambidentate character of the OPO ligand with inter­changeability of canonical structures having either 2-pyridinone or *N*-oxide electronic forms. In contrast with the four known alkyl *R*_2_Si(OPO)_2_ complexes in the crystalline state which favored primarily the ON-*trans*-ON isomer, the aryl derivative, Ph_2_Si(OPO)_2_, favored primarily the OC-*trans*-OC isomer and suggested that electron-withdrawing ancillary ligands might favor structures with primarily *N*-oxide forms. We report here the crystal structures and solution characterization of three additional aryl-substituted *R*_2_Si(OPO)_2_ [*R* = C_6_F_5_ (**1**), *p*-tolyl (**2**), mesityl (**3**)] complexes. 
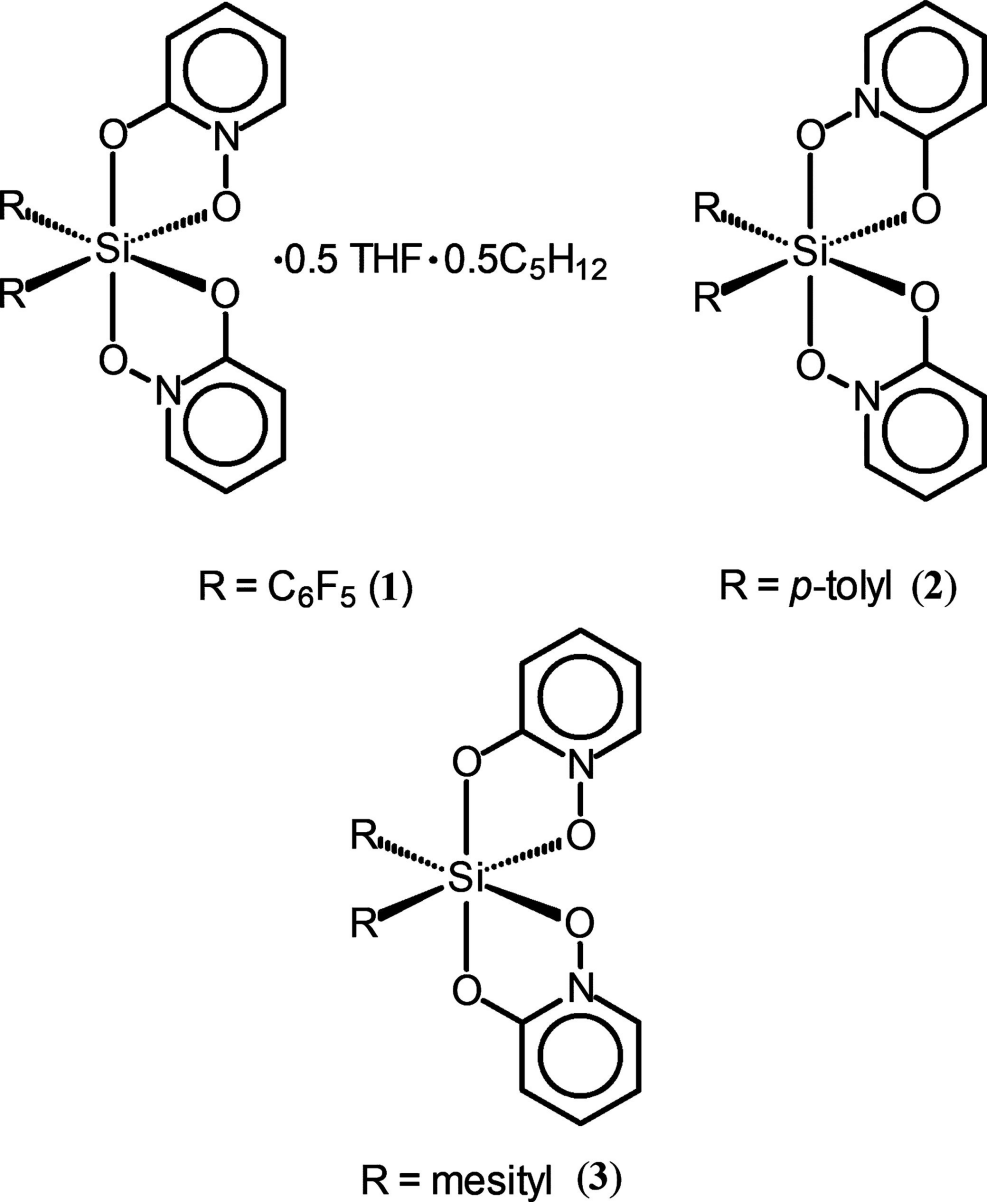


## 2. Structural commentary

**Figure 1:**
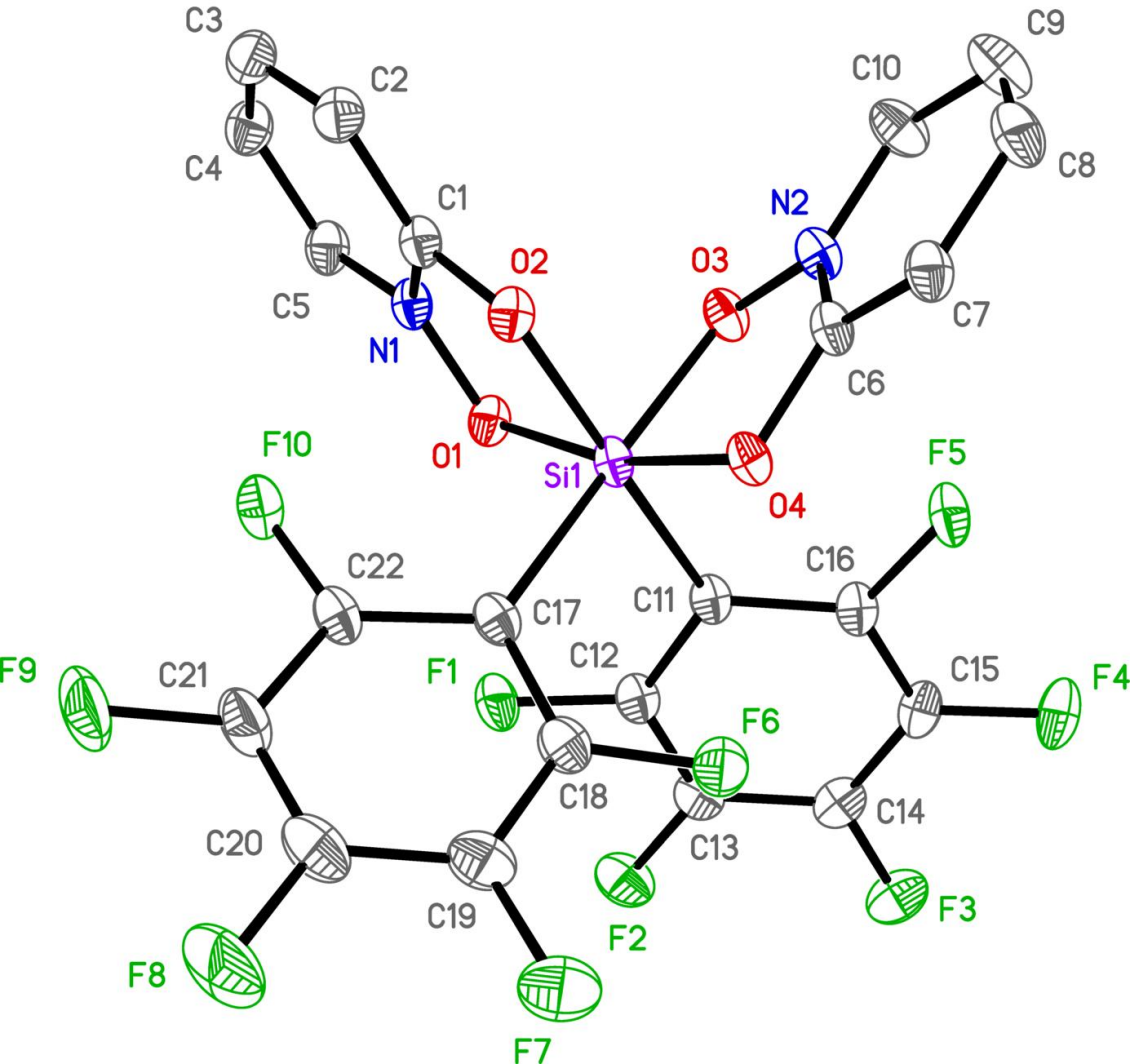
Anisotropic displacement ellipsoid plot of **1** drawn at the 50% probability level with H atoms and solvent omitted. Only the major components of disorder are shown.

There is one silicon complex in a general position per asymmetric unit for all three structures. In **1**, there are also solvents of crystallization (see *Refinement*). Each of the three complexes is hexa­coordinate in a distorted octa­hedral geometry with *cis*-aryl groups and two chelating OPO ligands (Figs. 1 ▸–3 ▸ ▸). Selected bond lengths and angles are summarized in Tables 1 ▸, 2 ▸ and 3 ▸. In all three complexes, the oxygen-bonded C and N atoms of each pyridine ring are modeled as disordered (see *Refinement*), which indicates the presence of up to three possible diastereomers in each. In **1**, the C1/N1 and C6/N2 disorder ratios indicate approximately equal C/N atom occupancy in both OPO ligand sites. In **2**, to an uncertain degree, a larger proportion of the ON-*trans*-ON arrangement is indicated from the disorder ratios, and in **3**, a larger proportion of the OC-*trans*-OC arrangement is indicated. In our previous work (Kraft & Brennessel, 2014 ▸), similarly disordered dialkyl *R*_2_Si(OPO)_2_ [*R* = Me, Et, *i*Pr; *R*_2_ = (CH_2_)_3_] complexes were found to favor a larger proportion of the ON-*trans*-ON arrangement, whereas the more electron-withdrawing Ph_2_Si(OPO)_2_ favored a larger proportion of the OC-*trans*-OC arrangement. The structures of **1**, **2**, and **3** indicate no trend in major isomer preference with ar­yl/electron withdrawing ancillary ligands. As in all other *R*_2_Si(OPO)_2_ complexes, the Si—O bonds *trans* to alkyl or aryl groups in **1**–**3** are consistently longer than those *cis*.

The ^29^Si NMR spectrum of **1** in DMSO-*d*_6_ displays a single broadened resonance at −152.5 ppm, consistent with hexa­coordinated silicon. Two sets of sharp OPO ligand resonances in 1:1 ratio are observed in the ^13^C NMR spectrum, and two sets of C_6_F_5_ ligand resonances in 1:1 ratio are observed in the ^19^F NMR spectrum, pointing to magnetic inequivalence of all four ligands. At 298 K, the *ortho* and *meta* ^19^F NMR resonances are significantly broadened, and each of the ten sharp OPO ligand ^13^C NMR resonances appears as a pair of closely-spaced peaks (a total of 20 peaks) separated by ≤ 0.2 ppm. Variable temperature NMR studies at 353 K show coalesced and sharpened *meta* ^19^F resonances, broadened *ortho* ^19^F resonances that approach coalescence, and ^1^H and ^13^C resonances of the OPO ligands that remain sharp. These observations are consistent with the absence of evidence of inter­conversion between diastereomers and the presence of two rotamers in 1:1 ratio of the totally asymmetric ON-*trans*-OC isomer with hindered rotation about the Si–C_6_F_5_ bonds. The absence of dynamic stereoisomerism at the observed temperatures is striking in light of that observed with all other known *R*_2_Si(OPO)_2_ complexes. This may be explained by the markedly stronger chelate inter­action in **1**, manifested by its shorter average Si—O bond lengths (Table 1 ▸) and larger O—Si—O ‘bite’ angles [84.60 (4) and 85.17 (4)°], which are ∼1–3° larger than those of all known *R*_2_Si(OPO)_2_ complexes (Kraft & Brennessel, 2014 ▸). As a result, Si←OC bond dissociation would be expected to be inhibited as observed, which has been shown as part of the mechanism of isomerization of *R*_2_Si(OPO)_2_ complexes. Similarly, inter­conversion of *fac* and *mer* isomers in the even more strongly chelated [Si(OPO)_3_]^+^ cation is not observed for likely the same reason (Kraft *et al.*, 2015 ▸). Bite angles in homoleptic [Si(OPO)_3_]^+^ silyl cations range from 87.0–87.4° in [Si(OPO)_3_]Cl·2CDCl_3_, [Si(OPO)_3_]Cl·*x*CH_3_CN, and [Si(OPO)_3_]·[CF_3_SO_3_]·0.5HOPO [Cambridge Structural Database (CSD; Groom *et al.*, 2016 ▸), version 5.45, update Nov. 2023; refcodes RUTQUU, RUTRAB (Kraft *et al.*, 2015 ▸) and QOXSIF (Tacke, Willeke, & Penka, 2001 ▸)], respectively, indicating even stronger chelate inter­actions in comparison with **1**. The presence of only one isomer of **1** in solution is consistent with the crystallographic data having a common disorder ratio of 0.52 (2):0.48 (2) for both C1/N1 and C6/N2. The ON-*trans*-OC isomer and mol­ecular superimposition of the flip of itself (*i.e.*, a *C*_2_ rotation about the axis bis­ecting the C—Si—C angle) uniquely reverses the positions of C and N atoms in all four oxygen-bonded sites, necessarily resulting in an equal disorder ratio.

The strength of the chelate inter­action increases in the complexes in the order **3**→**2**→**1** as given by decreasing average Si—O bond distances and increasing O_2_Si bite angles (Tables 1 ▸, 2 ▸ and 3 ▸). This can be explained by the electron-withdrawing effect of the fluoroaryl groups which strengthens the inter­action in **1** and the increase in steric hindrance from *ortho*-methyl substitution, which weakens the inter­action in **3**. Steric influences in **3** are further evident by the greater deviation of the *trans*-O—Si—O angle [162.48 (8)°] from ideal (*i.e.*, 180°) *versus* those in **1** and **2** [166.74 (4) and 165.96 (7)°, respectively] and by the larger C—Si—C angle in **3** *versus* **2** and **1**. The electron-donating *p*-tolyl groups of **2** appear to increase slightly the chelate strength of the OPO ligand in comparison with that in Ph_2_Si(OPO)_2_ given by the comparable Si—O bond lengths and ∼1° larger O_2_Si bite angles [for Ph_2_Si(OPO)_2_: Si—O = 1.9175 (4), 1.8157 (13) Å; O—Si—O = 82.47 (6)°].

For **2** in CDCl_3_ solution, a single set of OPO and *p*-tolyl ligand resonances was observed by ^1^H and ^13^C NMR spectroscopy with varying extents of broadened OPO ligand and *p*-tolyl peaks that sharpen further at higher temperature. These observations are consistent with stereodynamic isomerization occurring similar to that observed with Ph_2_Si(OPO)_2_ (Kraft & Brennessel, 2014 ▸). Complex **3** could not be characterized in solution due to its poor solubility.

Each O_2_Si chelate ring and planar OPO ligand in **1** forms a relatively large dihedral angle [9.60 (2) and 16.36 (4)°] in comparison with those of other alkyl *R*_2_Si(OPO)_2_ complexes [*R* = Me, Et, *i*Pr, *t*Bu; *R*_2_ = (CH_2_)_3_, range = 1.78–12.47°), **2** [2.41 (8) and 0.97 (9)°], and **3** [6.68 (11) and 8.41 (9)°]. Larger dihedral angles [both 21.51 (9)°] are also observed in Ph_2_Si(OPO)_2_. Unspecific crystal packing effects are likely responsible for these variations as no correlation could be found relating the magnitude of these fold angles with chelate strength or other ancillary ligand characteristics.

## 3. Supra­molecular features

**Figure 4:**
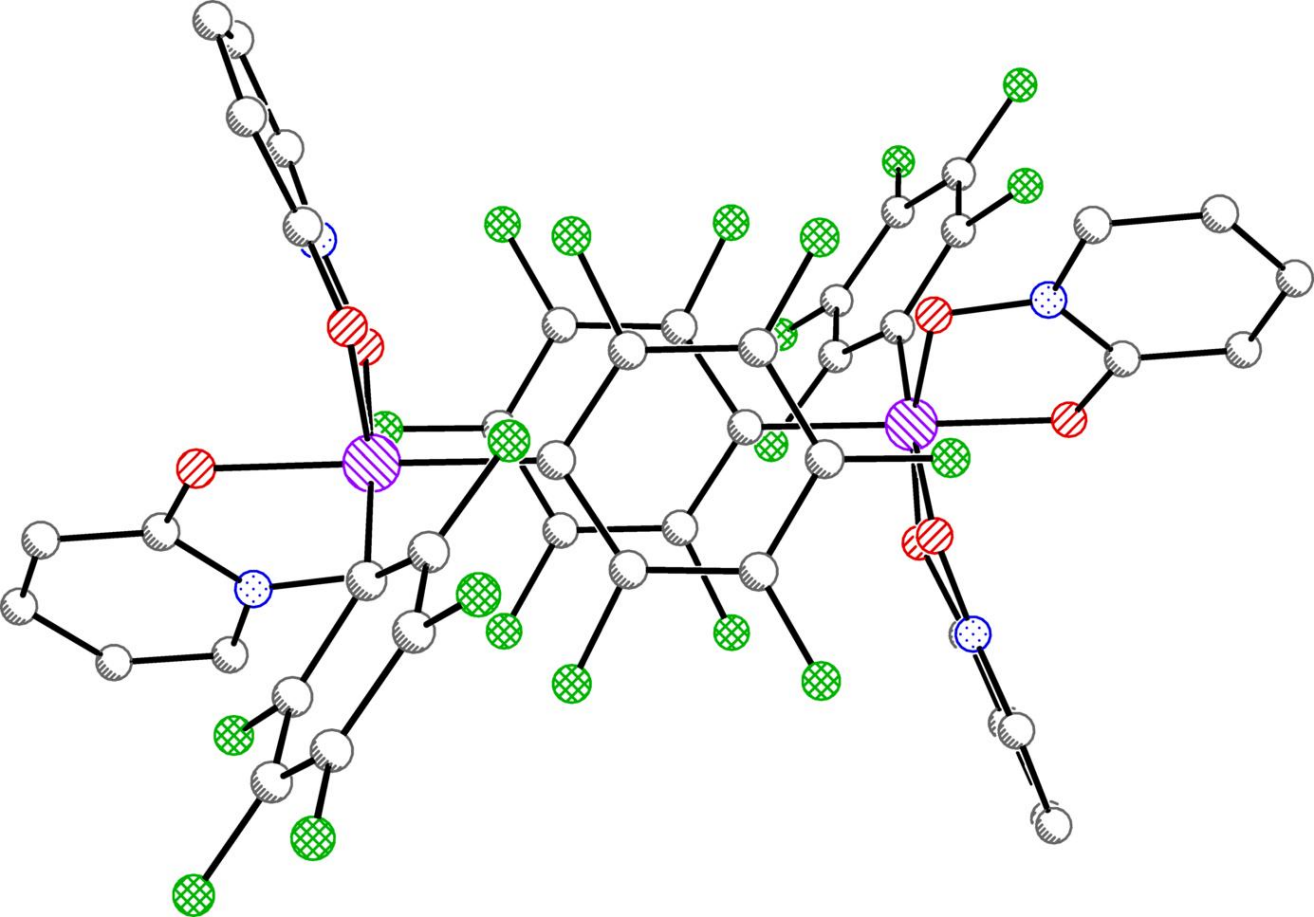
Offset parallel π–π inter­action between inverted pairs of mol­ecules of **1**. The second mol­ecule is generated by the symmetry operation 1 − *x*, 1 − *y*, 1 − *z*. Centroid–centroid distance, 3.86 Å.

In **1** there is an offset parallel π–π inter­action between ring C11–C16 from pairs of inverted mol­ecules (Fig. 4 ▸), with a centroid–centroid distance of 3.8613 (8) Å and an inter­planar distance of 3.7876 (13) Å. Further π–π inter­actions may have been inhibited during crystal growth by the presence of solvent. There are a few short inter­molecular C—H⋯F—C(aromatic) contacts, the strongest of which are listed in Table 4 ▸. However, it should be noted that only two [C2—H2⋯F1( 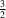 − *x*, 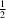 + *y*, 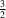 − *z*)] and C10—H10⋯F8(−1 + *x*, *y*, *z*)] have H⋯F distances of significance compared with the sum of the individual van der Waals radii (2.56 Å; Rowland & Taylor, 1996 ▸) and that these attractions tend to be very weak – of the order of the energies of van der Waals complexes (Howard *et al.*, 1996 ▸).

**Figure 5:**
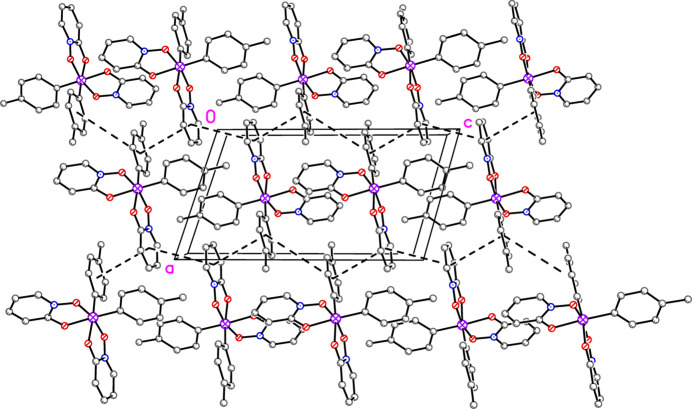
Packing plot of **2** with H atoms omitted. Rows of inter­locking mol­ecules along the [001] direction create two-dimensional sheets. Centroid–centroid distances are 3.76, 4.17, and 5.05 Å, for which the smaller two may allow for offset parallel π–π inter­actions.

**Figure 7:**
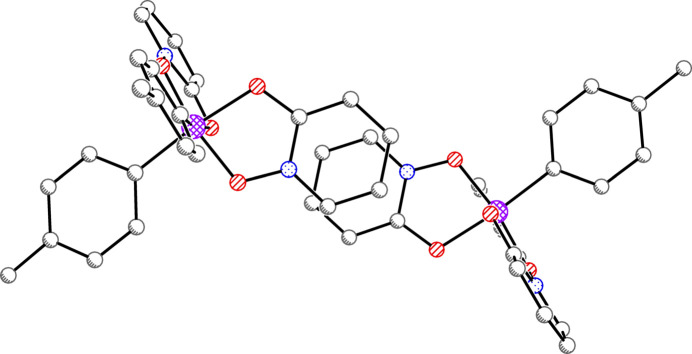
The sheets depicted in Figs. 5 ▸ and 6 ▸ are connected *via* additional π–π inter­actions between inverted pairs of mol­ecules. Second mol­ecule generated by 1 − *x*, −*y*, 1 − *z*. Centroid–centroid distance, 3.51 Å.

The packing of **2** features sheets of mol­ecules parallel to the *ac* plane (Figs. 5 ▸ and 6 ▸). Inverted pairs of ring N1/C1–C5 alternate with inverted pairs of ring C11–C16 to form staggered, but parallel arene ring alignments along [001] (Fig. 5 ▸). The centroid–centroid distances are 3.7548 (14), 4.1725 (12), and 5.0523 (13) Å with inter­planar spacings of 3.588 (2), 3.556 (3), and 3.532 (4) Å, respectively. The alignment of rings at the largest centroid-centroid distance of 5.05 Å is likely a mere consequence of a favorable packing arrangement rather than significant π–π overlap. These sheets are linked in the third dimension by pairs of offset parallel π–π inter­actions involving ring N2/C6–C10 (Fig. 7 ▸) with a centroid-centroid distance of 3.5067 (14) Å and an inter­planar spacing of 3.350 (2) Å.

**Figure 8:**
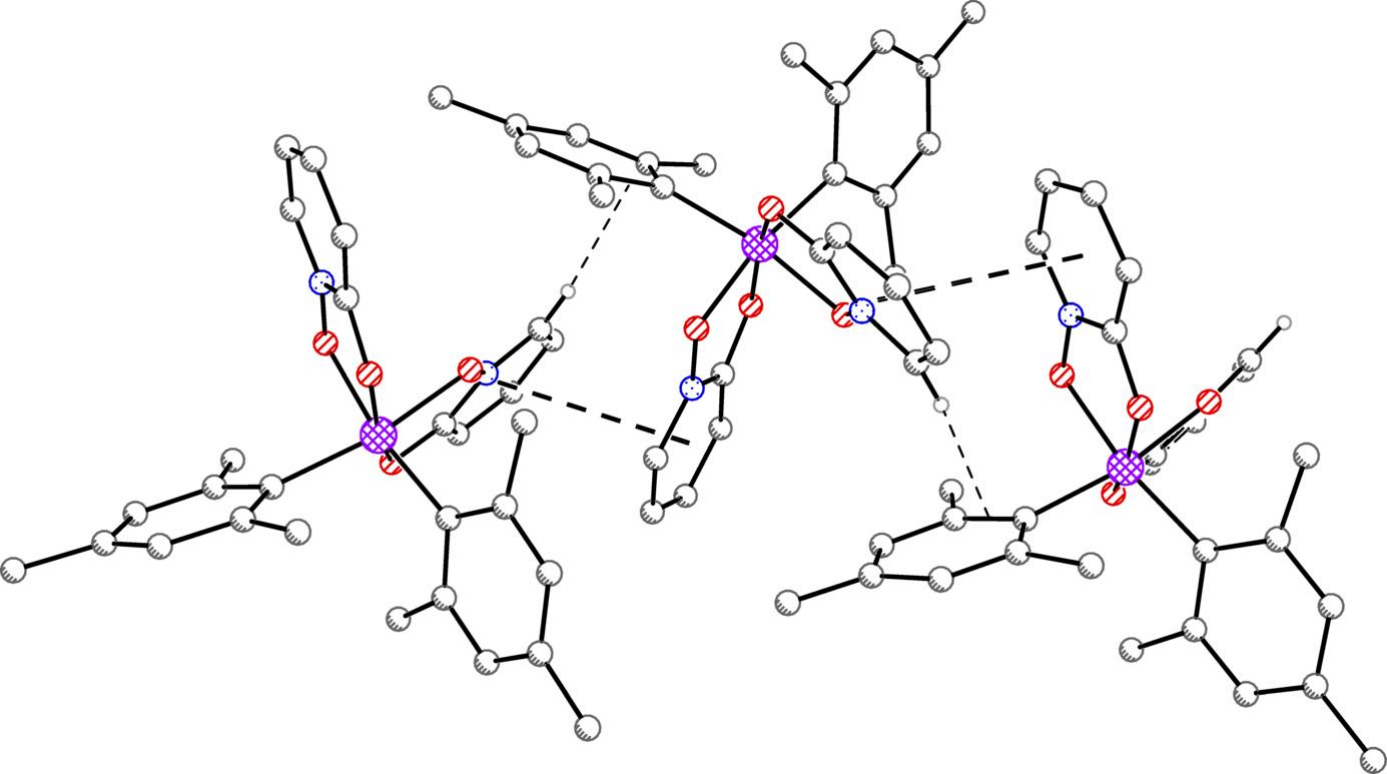
Possible π–π inter­action in **3** shown by thick dashes. Centroid–centroid distances, 3.74 Å. Angles between ring planes, 23°. Edge-to-face C—H⋯π inter­actions shown by thin dashes between H atoms and the π systems at the edge of each acceptor ring. Symmetry-equivalent mol­ecules generated by 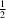 + *x*, 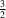 − *y*, 1 − *z* and 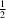 + *x*, 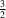 − *y*, 1 − *z*.

Mol­ecules of **3** appear linked along [100] *via* π–π inter­actions between rings N1/C1–C5 and N2/C6–C10 of symmetry-equivalent mol­ecules (Fig. 8 ▸). Although the centroid-centroid distance is short at 3.7416 (14) Å, the angle between ring planes is 23.03 (11)°, perhaps limiting the attractive force. The inter­planar spacings range from 3.191 (3) to 4.268 (3) Å, with an average of 3.722 (7) Å. One C—H⋯π inter­action accompanies each π–π inter­action just described (Fig. 8 ▸). The distance between H and the midpoint of the C11—C16 bond is 2.50 Å, with a C—H⋯CC(midpoint) angle of 174°. The angle between the plane containing the C—H donor and that of the π-acceptor is 68.27 (7)°.

## 4. Database survey

There are currently no reported structures of hexa­coordinate bis­(penta­fluoro­phen­yl)silicon(IV) complexes, nor other hexa­coordinate dimesit­ylsilicon(IV) complexes. The related hexa­coordinate pyri­thione (OPTO) complex, (*p*-tol­yl)_2_Si(OPTO)_2_, crystallizes with *cis* aryl groups and primarily with two bidentate OPTO ligands in an S-*trans*-S arrangement with additional disordered monodentate modes (CSD refcode DEWGAR; Tiede *et al.*, 2022 ▸). Mesit­yl_2_Si(OPTO)_2_ is tetra­coordinate with two monodentate *κO* OPTO ligands (CSD refcode DEWSUX; Tiede *et al.*, 2022 ▸).

There are five entries of hexa­coordinate *R*_2_Si(OPO)_2_ [*R* = Me, Et, *i*Pr, Ph; *R*_2_ = (CH_2_)_3_] complexes containing two bidentate OPO ligands (CSD refcodes NITSAM, NITSEQ, NITSOA, NISMIN, NITSUG, respectively; Kraft & Brennessel, 2014 ▸). Also reported with two bidentate OPO ligands are monoorgano neutral hexa­coordinate complexes, *R*Si(OPO)_2_ *X* (*X* = Cl, F; CSD refcodes ODEFIP, ODEFOV, ODEFUB, ODEHAJ, and ODEHEN), and cationic penta­coordinate complexes, [*R*Si(OPO)_2_]^+^ *X* ^−^ (*X* = Cl, tri­fluoro­methane­sulfonate; CSD refcodes ODEGAI, ODEGIQ, ODEGOW, and ODEGUC; Koch *et al.*, 2017 ▸). Other related entries include [Si(OPO)_2_(μ-CH_2_CH_2_SCH_2_C(=O)O)]_2_·2CH_3_CN and [O(CH_2_)_3_]Si(OPO)_2_ (CSD refcodes UBUWET and UBUWIX, respectively; Tacke, Burschka *et al.*, 2001 ▸). Monodentate OPO ligand complexes of any metal are limited to three organosilicon complexes: Me_3_Si(OPO), *t*Bu_2_Si(*κ* ^1^-OPO)(*κ* ^2^-OPO), and Ph_3_Si(OPO)·Ph_3_Si(OH)·0.5C_5_H_12_ (CSD refcodes NITROZ, NITSOA, and NITRIT, respectively; Kraft & Brennessel, 2014 ▸). Upon review of a total of 70 complexes of any metal in the CSD containing the OPO ligand (Groom *et al.*, 2016 ▸), complexes with OPO ligand/O_2_ *M* dihedral angles deviating more than 15° from coplanarity are relatively rare comprising of seven complexes of Si, V, Cu, Zn, Eu, Gd, and Th (CSD refcodes NISMIN: Kraft & Brennessel, 2014 ▸; OJEHOB: Jakusch *et al.*, 2010 ▸; HUSHEJ: Peyroux *et al.*, 2009 ▸; TADXAY: Puerta & Cohen, 2003 ▸; JAFZEW and JAFZIA: Tedeschi *et al.*, 2003 ▸; BURPEJ: Casellato *et al.*, 1983 ▸).

## 5. Synthesis and crystallization

(C_6_F_5_)_2_Si(OPO)_2_·0.5THF·0.5C_5_H_12_ (**1**): To a solution of HOPO (0.1508 g, 1.357 mmol) in ∼2 ml of THF was added a solution of (C_6_F_5_)_2_Si(OCH_3_)_2_ (0.2883 g, 1.025 mmol) in ∼2 ml THF. The resulting solution was stirred for two days and the solvent removed under vacuum. A portion (0.100 g) was recrystallized by vapor diffusion of *n*-pentane into a THF solution to yield white crystals of (C_6_F_5_)_2_Si(OPO)_2_·0.5THF·0.5C_5_H_12_. Subsequent washing of the crystals with THF and drying for 3 h under vacuum resulted in partial removal of solvents of crystallization, which analyzed as (C_6_F_5_)_2_Si(OPO)_2_·0.36C_4_H_8_O·0.11C_5_H_12_ (0.046 g, 46%) by a qu­anti­tative ^1^H NMR experiment and by elemental analysis. ^1^H NMR (DMSO-*d*_6_, 353 K): δ 0.87 (*t*, penta­ne), 1.28 (penta­ne), 1.77 (THF), 3.62 (THF), 7.10 (*m*, 3H), 7.35 (*ddd*, ^3^ *J* = 8.6, ^3^ *J* = 4.5, ^4^ *J* = 1.0 Hz, 1H), 7.88 (*m*, 2H), 8.41 (*ddd*, ^3^ *J* = 10.6, ^3^ *J* = 6.6, ^4^ *J* = 1.2 Hz, 1H), 8.64 (*m*, 1H). ^13^C NMR (DMSO-*d*_6_, 298 K): δ 13.9 (penta­ne), 21.7 (penta­ne), 25.1 (THF), 33.5 (penta­ne), 67.0 (THF), 112.0, 112.2, 112.2, 114.2, 114.2, 115.5, 115.5, 124.4 (*br*, Si—C), 132.6, 132.6, 132.7, 132.8, 136.0 (*br d*, ^1^ *J*_C—F_ = 250 Hz), 138.8 (*br d*, ^1^ *J*_C—F_ = 250 Hz), 138.9, 138.9, 139.8, 139.9, 147.7 (*br d*, ^1^ *J*_C—F_ = 230 Hz), 154.5, 154.6, 155.4 (CO), 155.4 (CO). ^19^F NMR (DMSO-*d*_6_, 298 K, referenced to α,α,α-tri­fluoro­toluene at δ −63.73): δ −167.1 (*br*, *m*-C_6_F_5_), −166.6 (*br*, *m*-C_6_F_5_), −160.8 (*m*, *p*-C_6_F_5_), −160.5 (*t*, *J* = 21.1 Hz, *p*-C_6_F_5_), −136.2 (*br*, *o*-C_6_F_5_), −130.0 (*br*, *o*-C_6_F_5_), −128.8 (*br*, *o*-C_6_F_5_). ^29^Si NMR (DMSO-*d*_6_, 298 K): δ −152.5 (*br*). Analysis calculated for (C_6_F_5_)_2_Si(OPO)_2_ 0.36·C_4_H_8_O 0.11·C_5_H_12_: C, 46.72%; H, 1.98%; N, 4.55%. Found: C, 47.09%; H, 1.95%; N, 4.68%.

*p*-Tol­yl_2_Si(OPO)_2_ (**2**): To a solution of Me_3_Si(OPO) (0.1243 g, 0.678 mmol) in 7 ml of CH_3_CN was added dropwise a solution of *p*-tol­yl_2_SiCl_2_ (87.0 µ*L*, *d* = 1.10 g ml^−1^, 0.340 mmol) in 2 ml of CH_3_CN at room temperature. The mixture was allowed to stand undisturbed for nine days. Decantation, washing with ∼1 ml of CH_3_CN, and drying under vacuum afforded 0.1132 g (75.5%) of a combination of a white powder and crystals used for structure determination. ^1^H NMR (CDCl_3_, 333 K): δ 2.24 (*s*, 6H), 6.61 (*m*, 2H), 6.82 (*br d*, ^3^ *J* = 7.9 Hz, 2H), 6.96 (*d*, ^3^ *J* = 7.8 Hz, 4H, *p*-tol­yl), 7.38 (*ddd*, ^3^ *J* = 7.3, ^3^ *J* = 8.7, ^4^ *J* = 1.7 Hz, 2H), 7.53 (*d*, ^3^ *J* = 7.8 Hz, 4H, *p*-tol­yl), 8.00 (*br d*, ^3^ *J* = 6.1 Hz, 2H). ^13^C NMR (CDCl_3_, 333 K): δ 21.4 (CH_3_), 111.5 (*br*), 113.2, 127.5, 132.4, 134.8, 135.1, 136.5 (*br*), 148.4 (*br*), 156.8 (CO). ^29^Si NMR (CDCl_3_, 333 K): δ −128.3. Analysis calculated for C_24_H_22_N_2_O_4_Si: C, 66.95; H, 5.15; N, 6.51. Found: C, 66.30; H, 5.09; N, 6.71.

Mesit­yl_2_Si(OPO)_2_ (**3**): To a filtered solution of Me_3_Si(OPO) (0.0904 g, 0.493 mmol) in 4 ml of CH_3_CN was added a filtered solution of mesit­yl_2_SiCl_2_ (0.0832 g, 0.247 mmol) in 4 ml of CH_3_CN. Colorless crystals deposited after one day at room temperature. Decantation and drying under vacuum afforded 0.0633 g (52.8%) of product that was insoluble in hot chloro­form and hot aceto­nitrile. An attempt to dissolve **3** in DMSO-*d*_6_ with heating resulted in dissolution with complete decomposition into unidentified products. NMR analysis of a CDCl_3_ solution prior to precipitation showed severely broadened indecipherable peaks. Analysis calculated for C_28_H_30_N_2_O_4_Si: C, 69.11; H, 6.21; N, 5.76. Found: C, 68.85; H, 6.16; N, 5.69.

## 6. Refinement

Crystal data, data collection and structure refinement details are summarized in Table 5 ▸. In all three structures, both bidentate ligands are disordered with the coplanar flips of themselves. For the rings containing C1/N1 and C6/N2, respectively, the disorder ratios are 0.52 (2):0.48 (2) and 0.52 (2):0.48 (2), 0.66 (2):0.34 (2) and 0.61 (2):0.39 (2), and 0.68 (3):0.32 (3) and 0.61 (3):0.39 (3), for structures **1**, **2**, and **3**, respectively. Due to resolution limitations, the disorder model did not include the entire ring, but was modeled by refining the occupancies of the two atoms types (C and N) at the oxygen-coordinating portions of the rings. The occupancies at each site were constrained to sum to one and additionally to sum to one C and one N atom between the two sites on each ring. The positional and anisotropic displacement parameters, respectively, at each site of disorder were constrained to be equivalent. It is understood that this type of disorder model will likely exhibit a weighted average of Si—O bond lengths, trending with the disorder ratios.

In **1**, the solvent volume contains one each of THF and *n*-pentane disordered over a crystallographic inversion center (0.50:0.50). Analogous bond lengths and angles in both directions along each solvent mol­ecule were restrained to be similar. Anisotropic displacement parameters for proximal atoms were restrained to be similar.

All H atoms were placed geometrically and treated as riding atoms. Aromatic/*sp* ^2^, C–H = 0.95 Å and methyl­ene, C–H = 0.99 Å, with *U*_iso_(H) = 1.2*U*_eq_(C). Methyl, C–H = 0.98 Å, with *U*_iso_(H) = 1.5*U*_eq_(C).

For **1** the maximum residual peak of 0.61 e^−^ Å^−3^ and the deepest hole of −0.58 e^−^ Å^−3^ are found 0.69 and 0.35 Å from atoms C21 and C25, respectively.

For **2** the maximum residual peak of 1.01 e^−^ Å^−3^ and the deepest hole of −0.43 e^−^ Å^−3^ are found 0.92 and 0.61 Å from atom Si1.

For **3** the maximum residual peak of 0.27 e^−^ Å^−3^ and the deepest hole of −0.25 e^−^ Å^−3^ are found 0.92 and 0.58 Å from atoms C20 and Si1, respectively.

## Supplementary Material

Crystal structure: contains datablock(s) 1, 2, 3, global. DOI: 10.1107/S2056989024001543/ee2004sup1.cif

Structure factors: contains datablock(s) 1. DOI: 10.1107/S2056989024001543/ee20041sup2.hkl

Structure factors: contains datablock(s) 2. DOI: 10.1107/S2056989024001543/ee20042sup3.hkl

Structure factors: contains datablock(s) 3. DOI: 10.1107/S2056989024001543/ee20043sup4.hkl

CCDC references: 2333179, 2333178, 2333177

Additional supporting information:  crystallographic information; 3D view; checkCIF report

## Figures and Tables

**Figure 2:**
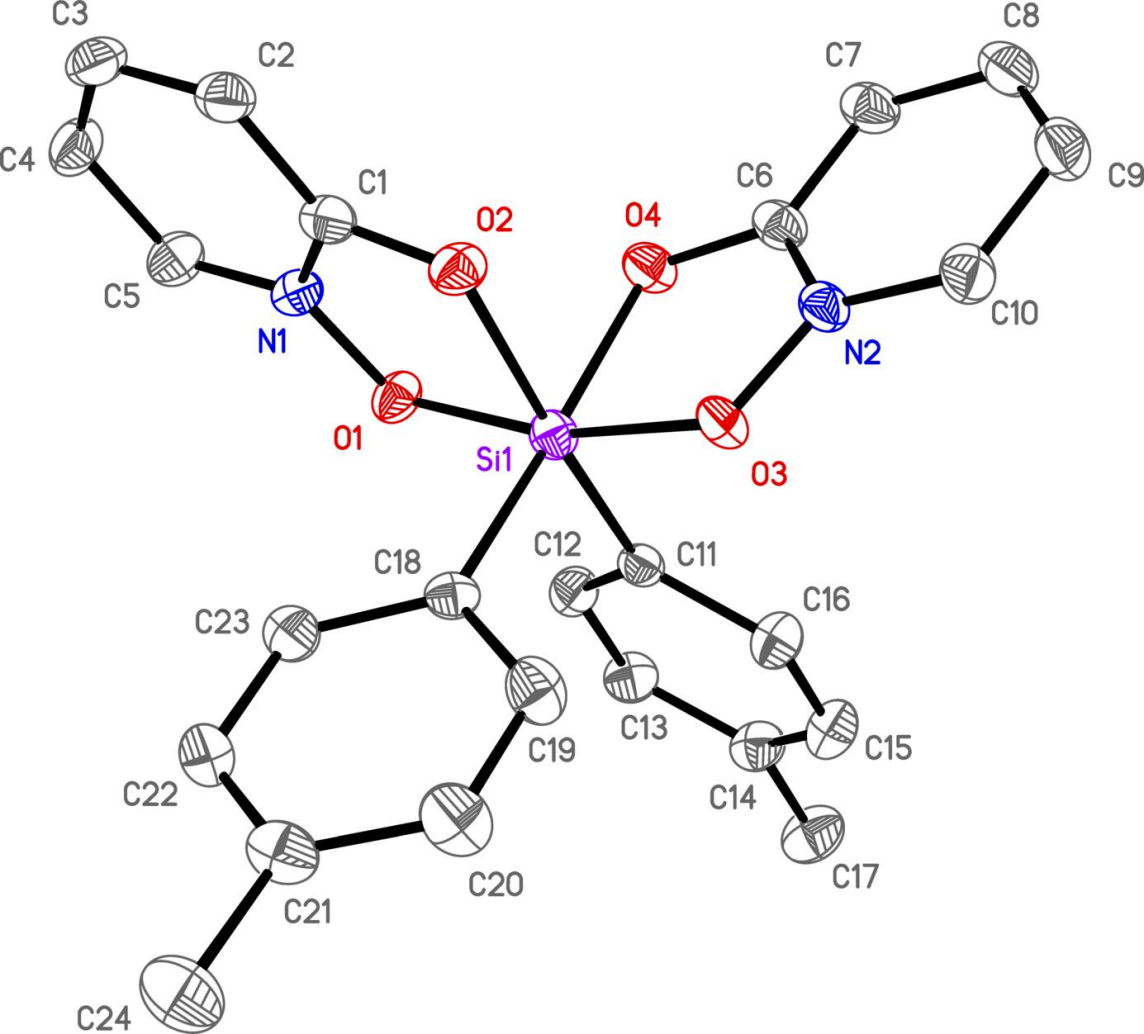
Anisotropic displacement ellipsoid plot of **2** drawn at the 50% probability level with H atoms omitted. Only the major components of disorder are shown.

**Figure 3:**
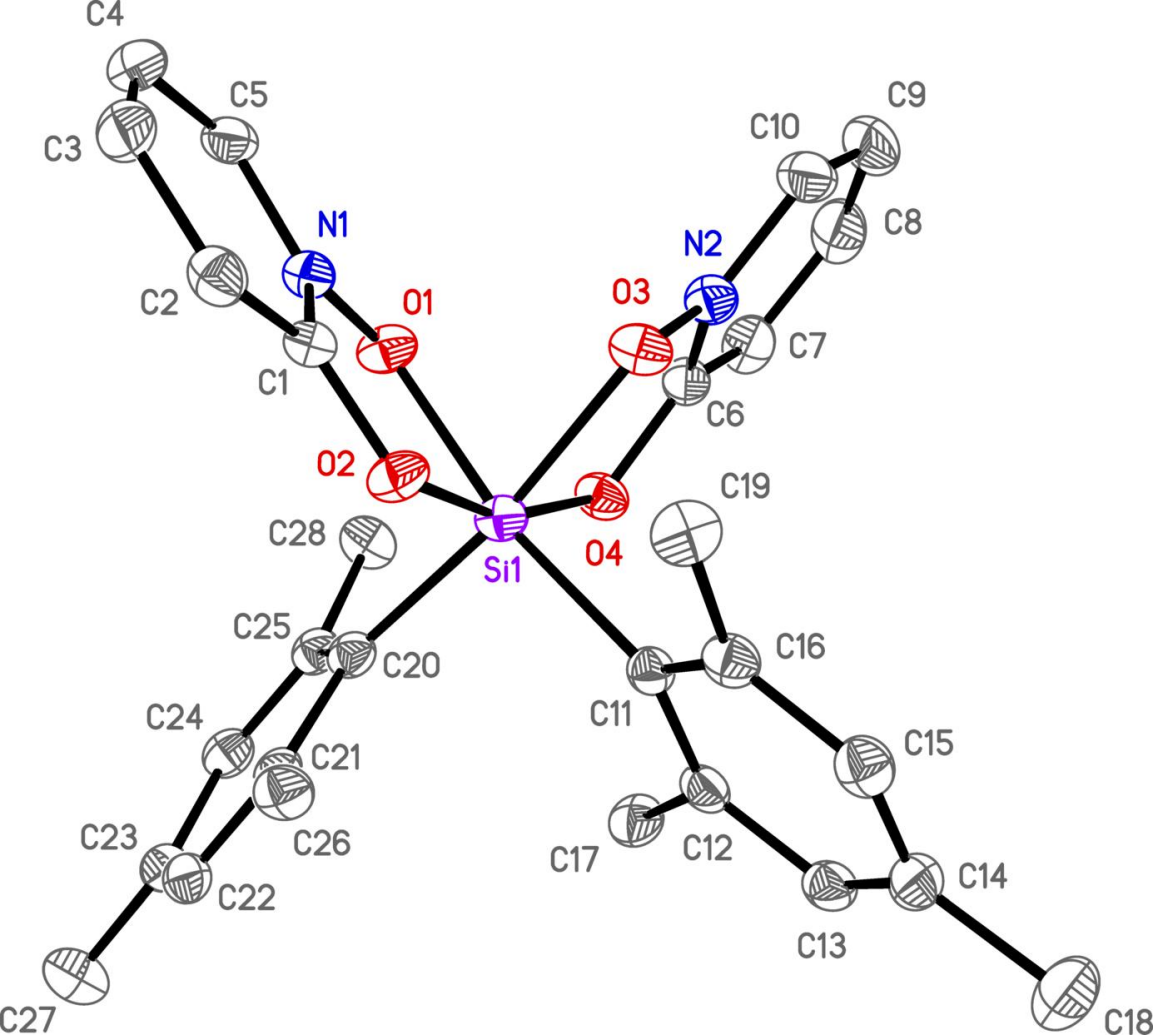
Anisotropic displacement ellipsoid plot of **3** drawn at the 50% probability level with H atoms omitted. Only the major components of disorder are shown.

**Figure 6:**
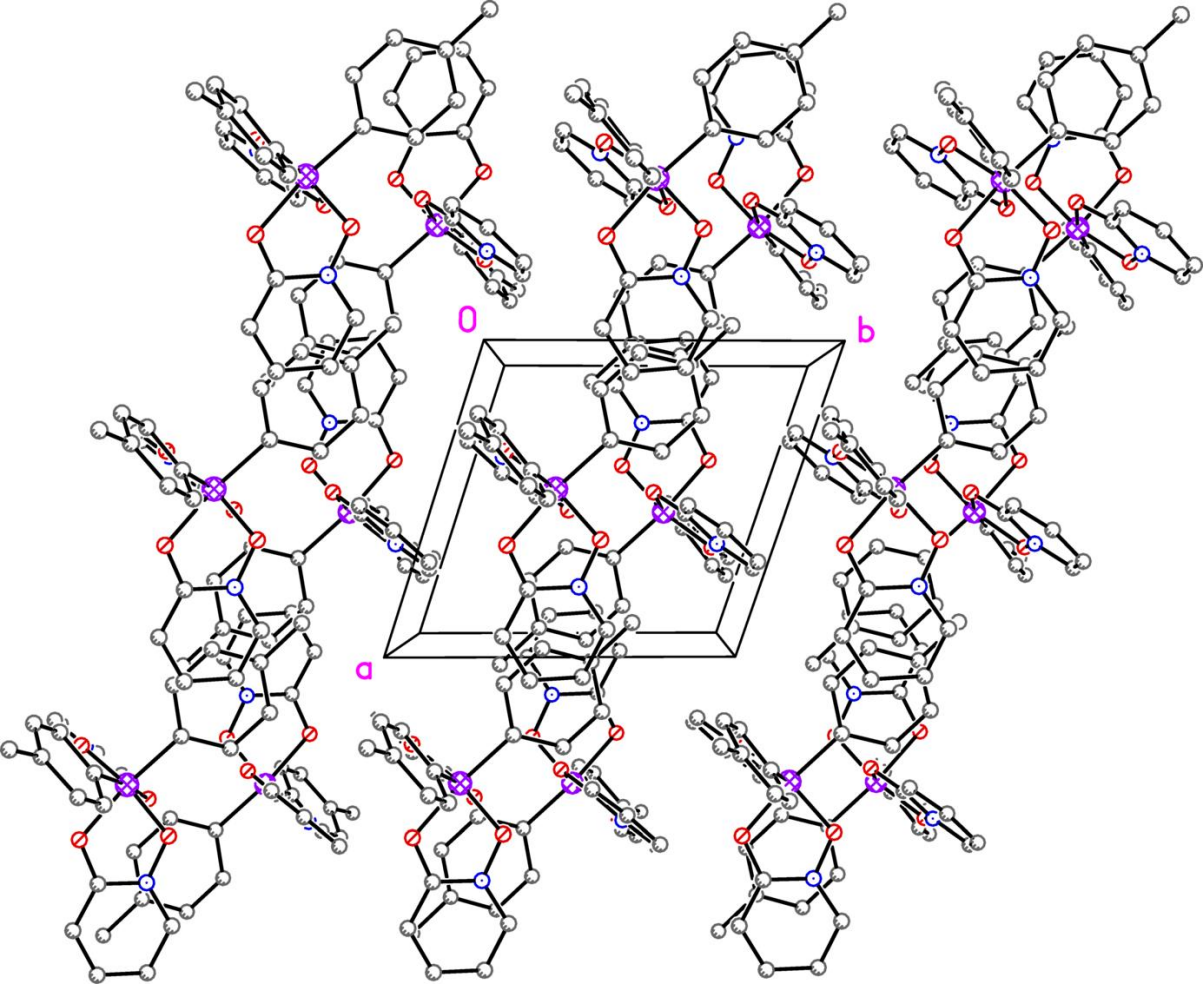
Packing plot of **2** with H atoms omitted that shows the divisions between the sheets shown in Fig. 5 ▸.

**Table 1:** Selected geometric parameters (Å, °) for **1** [Chem scheme1]

Si1—O1	1.7910 (9)	Si1—O2	1.8503 (9)
Si1—O4	1.8042 (9)	Si1—C11	1.9559 (12)
Si1—O3	1.8480 (9)	Si1—C17	1.9683 (12)
			
O1—Si1—O4	166.74 (4)	O3—Si1—C11	90.16 (4)
O1—Si1—O3	86.65 (4)	O2—Si1—C11	175.99 (5)
O4—Si1—O3	84.60 (4)	O1—Si1—C17	99.44 (5)
O1—Si1—O2	85.17 (4)	O4—Si1—C17	88.68 (4)
O4—Si1—O2	84.53 (4)	O3—Si1—C17	172.66 (4)
O3—Si1—O2	87.56 (4)	O2—Si1—C17	88.88 (5)
O1—Si1—C11	91.41 (4)	C11—Si1—C17	93.75 (5)
O4—Si1—C11	98.54 (4)		

**Table 2:** Selected geometric parameters (Å, °) for **2** [Chem scheme1]

Si1—O3	1.8093 (14)	Si1—C11	1.9202 (19)
Si1—O1	1.8097 (14)	Si1—O2	1.9290 (15)
Si1—O4	1.9179 (15)	Si1—C18	1.9301 (19)
			
O3—Si1—O1	165.96 (7)	O4—Si1—O2	83.28 (6)
O3—Si1—O4	83.76 (6)	C11—Si1—O2	171.36 (8)
O1—Si1—O4	86.24 (7)	O3—Si1—C18	91.04 (7)
O3—Si1—C11	98.02 (8)	O1—Si1—C18	97.64 (8)
O1—Si1—C11	91.68 (7)	O4—Si1—C18	171.40 (7)
O4—Si1—C11	89.37 (7)	C11—Si1—C18	98.16 (8)
O3—Si1—O2	85.72 (6)	O2—Si1—C18	89.53 (7)
O1—Si1—O2	83.35 (6)		

**Table 3:** Selected geometric parameters (Å, °) for **3** [Chem scheme1]

Si1—O1	1.9291 (16)	Si1—O4	1.8096 (15)
Si1—O2	1.7896 (15)	Si1—C11	1.975 (2)
Si1—O3	1.9581 (16)	Si1—C20	1.955 (2)
			
O1—Si1—O3	80.99 (7)	O3—Si1—C11	88.61 (8)
O1—Si1—C11	169.59 (8)	O4—Si1—O1	84.30 (7)
O1—Si1—C20	90.09 (8)	O4—Si1—O3	81.82 (7)
O2—Si1—O1	83.25 (7)	O4—Si1—C11	94.57 (8)
O2—Si1—O3	84.09 (7)	O4—Si1—C20	95.73 (8)
O2—Si1—O4	162.48 (8)	C20—Si1—O3	170.93 (8)
O2—Si1—C11	95.44 (8)	C20—Si1—C11	100.32 (9)
O2—Si1—C20	96.57 (8)		

**Table 4:** Hydrogen-bond geometry (Å, °) for **1** [Chem scheme1]

*D*—H⋯*A*	*D*—H	H⋯*A*	*D*⋯*A*	*D*—H⋯*A*
C2—H2⋯F1^i^	0.95	2.34	3.2809 (15)	170
C4—H4⋯F6^ii^	0.95	2.68	3.5757 (16)	158
C5—H5⋯F4^iii^	0.95	2.59	3.2997 (15)	132
C7—H7⋯F5^iv^	0.95	2.57	3.2307 (14)	127
C8—H8⋯F6^iv^	0.95	2.56	3.2230 (15)	127
C10—H10⋯F8^v^	0.95	2.37	3.0797 (16)	131

Symmetry codes: (i) 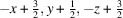 ; (ii) 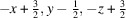 ; (iii) 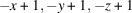 ; (iv) 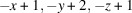 ; (v) 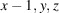 .

**Table 5:** Experimental details

	**1**	**2**	**3**
Crystal data			
Chemical formula	C_22_H_8_F_10_N_2_O_4_Si·0.5C_5_H_12_·0.5C_4_H_8_O	C_24_H_22_N_2_O_4_Si	C_28_H_30_N_2_O_4_Si
*M*_r_	654.52	430.52	486.63
Crystal system, space group	Monoclinic, *P*2_1_/*n*	Triclinic, *P* 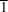	Orthorhombic, *P*2_1_2_1_2_1_
Temperature (K)	100	100	100
*a*, *b*, *c* (Å)	12.6809 (9), 12.1217 (9), 17.7335 (13)	8.5662 (8), 8.8343 (8), 14.7801 (14)	12.5710 (2), 12.68898 (19), 15.3580 (2)
α, β, γ (°)	90, 105.7674 (15), 90	93.057 (2), 105.3716 (19), 106.7565 (18)	90, 90, 90
*V* (Å^3^)	2623.3 (3)	1022.45 (17)	2449.80 (7)
*Z*	4	2	4
Radiation type	Mo *K*α	Mo *K*α	Cu *K*α
μ (mm^−1^)	0.20	0.15	1.15
Crystal size (mm)	0.40 × 0.36 × 0.14	0.24 × 0.24 × 0.20	0.09 × 0.07 × 0.06

Data collection			
Diffractometer	Bruker SMART APEXII CCD platform	Bruker SMART APEXII CCD platform	XtaLAB Synergy, Dualflex, HyPix
Absorption correction	Multi-scan (*SADABS*; Krause *et al.*, 2015 ▸)	Multi-scan (*SADABS*; Krause *et al.*, 2015 ▸)	Multi-scan (*CrysAlis PRO*; Rigaku OD, 2019 ▸)
*T*_min_, *T*_max_	0.694, 0.748	0.695, 0.746	0.674, 1.000
No. of measured, independent and observed [*I* > 2σ(*I*)] reflections	97594, 14595, 9977	25883, 6239, 4231	22120, 5138, 4847
*R*_int_	0.043	0.065	0.048
(sin θ/λ)_max_ (Å^−1^)	0.881	0.715	0.634

Refinement			
*R*[*F* ^2^ > 2σ(*F* ^2^)], *wR*(*F* ^2^), *S*	0.051, 0.163, 1.03	0.054, 0.149, 1.05	0.032, 0.079, 1.05
No. of reflections	14595	6239	5138
No. of parameters	446	284	324
No. of restraints	55	0	0
H-atom treatment	H-atom parameters constrained	H-atom parameters constrained	H-atom parameters constrained
Δρ_max_, Δρ_min_ (e Å^−3^)	0.61, −0.58	1.01, −0.44	0.27, −0.25
Absolute structure	–	–	Flack *x* determined using 1985 quotients [(*I* ^+^)−(*I* ^−^)]/[(*I* ^+^)+(*I* ^−^)] (Parsons et al., 2013 ▸)
Absolute structure parameter	–	–	−0.034 (17)

Computer programs: *APEX3* (Bruker, 2016 ▸), *SAINT* (Bruker, 2013 ▸), *CrysAlis PRO* (Rigaku OD, 2019 ▸), *SHELXT2014/5* and *SHELXT2018/2* (Sheldrick, 2015*a* ▸), *SHELXL2019/3* (Sheldrick, 2015*b* ▸), *SHELXTL* (Sheldrick, 2008 ▸), and *OLEX2* (Dolomanov *et al.*, 2009 ▸).

## supplementary crystallographic information

### Bis[1-oxopyridin-2-olato(1-)]bis(pentafluorophenyl)silicon(IV)–tetrahydrofuran–pentane (2/1/1) (1) Crystal data

**Table:**

C_22_H_8_F_10_N_2_O_4_Si·0.5C_5_H_12_·0.5C_4_H_8_O	*F*(000) = 1324
*M**_r_* = 654.52	*D*_x_ = 1.657 Mg m^−^^3^
Monoclinic, *P*2_1_/*n*	Mo *K*α radiation, λ = 0.71073 Å
*a* = 12.6809 (9) Å	Cell parameters from 3989 reflections
*b* = 12.1217 (9) Å	θ = 2.3–37.4°
*c* = 17.7335 (13) Å	µ = 0.20 mm^−^^1^
β = 105.7674 (15)°	*T* = 100 K
*V* = 2623.3 (3) Å^3^	Plate, colorless
*Z* = 4	0.40 × 0.36 × 0.14 mm

### Bis[1-oxopyridin-2-olato(1-)]bis(pentafluorophenyl)silicon(IV)–tetrahydrofuran–pentane (2/1/1) (1) Data collection

**Table:**

Bruker SMART APEXII CCD platform diffractometer	9977 reflections with *I* > 2σ(*I*)
Radiation source: fine-focus sealed tube	*R*_int_ = 0.043
ω scans	θ_max_ = 38.8°, θ_min_ = 1.8°
Absorption correction: multi-scan (SADABS; Krause *et al.*, 2015)	*h* = −22→22
*T*_min_ = 0.694, *T*_max_ = 0.748	*k* = −21→21
97594 measured reflections	*l* = −30→30
14595 independent reflections	

### Bis[1-oxopyridin-2-olato(1-)]bis(pentafluorophenyl)silicon(IV)–tetrahydrofuran–pentane (2/1/1) (1) Refinement

**Table:**

Refinement on *F*^2^	Primary atom site location: dual
Least-squares matrix: full	Secondary atom site location: difference Fourier map
*R*[*F*^2^ > 2σ(*F*^2^)] = 0.051	Hydrogen site location: inferred from neighbouring sites
*wR*(*F*^2^) = 0.163	H-atom parameters constrained
*S* = 1.03	*w* = 1/[σ^2^(*F*_o_^2^) + (0.0822*P*)^2^ + 0.9141*P*] where *P* = (*F*_o_^2^ + 2*F*_c_^2^)/3
14595 reflections	(Δ/σ)_max_ = 0.001
446 parameters	Δρ_max_ = 0.61 e Å^−^^3^
55 restraints	Δρ_min_ = −0.58 e Å^−^^3^

### Bis[1-oxopyridin-2-olato(1-)]bis(pentafluorophenyl)silicon(IV)–tetrahydrofuran–pentane (2/1/1) (1) Special details

**Table:**

Geometry. All esds (except the esd in the dihedral angle between two l.s. planes) are estimated using the full covariance matrix. The cell esds are taken into account individually in the estimation of esds in distances, angles and torsion angles; correlations between esds in cell parameters are only used when they are defined by crystal symmetry. An approximate (isotropic) treatment of cell esds is used for estimating esds involving l.s. planes.
Refinement. Both bidentate ligands are disordered with the coplanar flips of themselves (0.524 (16):0.476 (16) and 0.516 (15):0.484 (16) for the rings containing C1/N1 and C6/N2, respectively). Due to resolution limitations, the disorder was modeled by refining the occupancies of the two atoms types (C and N) at the oxygen-coordinating portions of the rings. The occupancies at each site were constrained to sum to one and additionally sum to one C and one N atom between the two sites on each ring. The positional and anisotropic displacement parameters,respectively, at each site of disorder were constrained to be equivalent.The solvent volume contains once each of n-pentane and tetrahydrofuran disordered over a crystallographic inversion center (0.50:0.50). Analogous bond lengths and angles in both directions along each solvent molecule were restrained to be similar. Anisotropic displacement parameters for proximal atoms were restrained to be similar.

### Bis[1-oxopyridin-2-olato(1-)]bis(pentafluorophenyl)silicon(IV)–tetrahydrofuran–pentane (2/1/1) (1) Fractional atomic coordinates and isotropic or equivalent isotropic displacement parameters (Å^2^)

**Table:**

	*x*	*y*	*z*	*U*_iso_*/*U*_eq_	Occ. (<1)
Si1	0.66487 (2)	0.71292 (2)	0.62708 (2)	0.01575 (6)	
O1	0.67613 (7)	0.58885 (7)	0.68356 (5)	0.01829 (14)	
O2	0.69747 (7)	0.78437 (7)	0.72284 (5)	0.01843 (14)	
O3	0.51949 (7)	0.71616 (6)	0.62790 (5)	0.01877 (15)	
O4	0.63848 (6)	0.84997 (7)	0.58666 (5)	0.01750 (14)	
N1	0.68205 (8)	0.60779 (8)	0.75861 (6)	0.01816 (19)	0.524 (16)
C1	0.69390 (9)	0.71546 (8)	0.78005 (6)	0.01806 (18)	0.524 (16)
N2	0.48096 (8)	0.81844 (9)	0.61909 (7)	0.01913 (19)	0.516 (15)
C6	0.54737 (8)	0.89276 (8)	0.59860 (6)	0.01757 (18)	0.516 (15)
N1'	0.69390 (9)	0.71546 (8)	0.78005 (6)	0.01806 (18)	0.476 (16)
C1'	0.68205 (8)	0.60779 (8)	0.75861 (6)	0.01816 (19)	0.476 (16)
N2'	0.54737 (8)	0.89276 (8)	0.59860 (6)	0.01757 (18)	0.484 (15)
C6'	0.48096 (8)	0.81844 (9)	0.61909 (7)	0.01913 (19)	0.484 (15)
C2	0.70444 (11)	0.74796 (10)	0.85565 (7)	0.0224 (2)	
H2	0.713350	0.823669	0.869789	0.027*	
C3	0.70189 (12)	0.66902 (12)	0.91066 (8)	0.0259 (2)	
H3	0.708483	0.689880	0.963383	0.031*	
C4	0.68956 (11)	0.55763 (11)	0.88890 (8)	0.0250 (2)	
H4	0.687662	0.503036	0.926946	0.030*	
C5	0.68013 (10)	0.52686 (10)	0.81226 (7)	0.0215 (2)	
H5	0.672500	0.451465	0.797136	0.026*	
C7	0.52081 (10)	1.00284 (9)	0.59067 (7)	0.02030 (19)	
H7	0.569884	1.054923	0.578717	0.024*	
C8	0.42148 (11)	1.03599 (10)	0.60042 (8)	0.0255 (2)	
H8	0.400870	1.111515	0.594537	0.031*	
C9	0.35103 (11)	0.95821 (12)	0.61899 (10)	0.0304 (3)	
H9	0.281838	0.980781	0.624646	0.036*	
C10	0.38159 (10)	0.84898 (11)	0.62913 (9)	0.0258 (2)	
H10	0.334779	0.795943	0.642792	0.031*	
C11	0.62534 (9)	0.62938 (9)	0.52904 (7)	0.01742 (18)	
C12	0.68528 (9)	0.53833 (9)	0.51616 (7)	0.01920 (19)	
F1	0.77010 (7)	0.50006 (6)	0.57439 (5)	0.02438 (15)	
C13	0.66631 (10)	0.48285 (10)	0.44545 (8)	0.0219 (2)	
F2	0.72793 (8)	0.39565 (7)	0.43788 (6)	0.03029 (18)	
C14	0.58391 (11)	0.51903 (10)	0.38166 (7)	0.0229 (2)	
F3	0.56808 (8)	0.47189 (7)	0.31140 (5)	0.03075 (18)	
C15	0.52011 (10)	0.60735 (10)	0.39152 (7)	0.0219 (2)	
F4	0.43903 (8)	0.64323 (7)	0.33081 (5)	0.03056 (18)	
C16	0.54109 (9)	0.65902 (9)	0.46376 (7)	0.01871 (18)	
F5	0.47407 (6)	0.74494 (6)	0.46711 (5)	0.02394 (15)	
C17	0.81897 (9)	0.73023 (9)	0.62649 (7)	0.01881 (18)	
C18	0.85009 (10)	0.78010 (10)	0.56525 (8)	0.0225 (2)	
F6	0.77384 (7)	0.80890 (7)	0.49856 (5)	0.02657 (16)	
C19	0.95760 (12)	0.80612 (13)	0.56729 (9)	0.0300 (3)	
F7	0.98118 (9)	0.85775 (10)	0.50685 (7)	0.0423 (2)	
C20	1.04070 (11)	0.77953 (15)	0.63276 (11)	0.0349 (3)	
F8	1.14520 (8)	0.80396 (12)	0.63564 (8)	0.0545 (3)	
C21	1.01591 (11)	0.72697 (13)	0.69452 (10)	0.0305 (3)	
F9	1.09644 (7)	0.70038 (10)	0.75876 (7)	0.0443 (3)	
C22	0.90737 (10)	0.70317 (10)	0.69006 (8)	0.0221 (2)	
F10	0.89124 (6)	0.65161 (7)	0.75357 (5)	0.02621 (16)	
C23	0.4695 (4)	0.9762 (6)	0.9416 (4)	0.086 (2)	0.5
H23A	0.417411	0.974458	0.973522	0.128*	0.5
H23B	0.511420	1.045096	0.951452	0.128*	0.5
H23C	0.519747	0.913386	0.955440	0.128*	0.5
C24	0.4109 (6)	0.9697 (6)	0.8603 (5)	0.0749 (18)	0.5
H24A	0.464327	0.975185	0.828792	0.090*	0.5
H24B	0.361469	1.034272	0.847065	0.090*	0.5
C25	0.3418 (6)	0.8635 (5)	0.8355 (4)	0.0589 (13)	0.5
H25A	0.296849	0.849535	0.872273	0.071*	0.5
H25B	0.291733	0.873160	0.782364	0.071*	0.5
C26	0.4188 (6)	0.7637 (8)	0.8360 (5)	0.0513 (16)	0.5
H26A	0.457846	0.775760	0.795365	0.062*	0.5
H26B	0.474409	0.761122	0.887388	0.062*	0.5
C27	0.3620 (4)	0.6549 (4)	0.8216 (3)	0.0601 (12)	0.5
H27A	0.325890	0.640356	0.862907	0.090*	0.5
H27B	0.415795	0.596735	0.821837	0.090*	0.5
H27C	0.307168	0.656125	0.770602	0.090*	0.5
O5'	0.4671 (2)	0.8125 (3)	0.79186 (18)	0.0468 (6)	0.5
C23'	0.4719 (4)	0.9254 (4)	0.8165 (3)	0.0516 (10)	0.5
H23D	0.483429	0.974883	0.775048	0.062*	0.5
H23E	0.532101	0.936716	0.864779	0.062*	0.5
C24'	0.3630 (5)	0.9471 (5)	0.8314 (4)	0.0565 (12)	0.5
H24C	0.368106	1.006523	0.870569	0.068*	0.5
H24D	0.307096	0.967374	0.782534	0.068*	0.5
C25'	0.3374 (6)	0.8395 (5)	0.8620 (4)	0.0616 (14)	0.5
H25C	0.257285	0.830186	0.852460	0.074*	0.5
H25D	0.372560	0.833615	0.918975	0.074*	0.5
C26'	0.3826 (7)	0.7550 (8)	0.8179 (6)	0.062 (2)	0.5
H26C	0.414208	0.692110	0.852291	0.075*	0.5
H26D	0.324670	0.726969	0.772589	0.075*	0.5

### Bis[1-oxopyridin-2-olato(1-)]bis(pentafluorophenyl)silicon(IV)–tetrahydrofuran–pentane (2/1/1) (1) Atomic displacement parameters (Å^2^)

**Table:**

	*U*^11^	*U*^22^	*U*^33^	*U*^12^	*U*^13^	*U*^23^
Si1	0.01361 (12)	0.01244 (12)	0.01899 (14)	−0.00038 (9)	0.00070 (10)	0.00039 (9)
O1	0.0211 (4)	0.0138 (3)	0.0178 (3)	0.0001 (3)	0.0015 (3)	0.0003 (3)
O2	0.0201 (3)	0.0138 (3)	0.0194 (3)	−0.0008 (3)	0.0020 (3)	0.0006 (3)
O3	0.0157 (3)	0.0129 (3)	0.0264 (4)	0.0001 (2)	0.0035 (3)	0.0010 (3)
O4	0.0140 (3)	0.0142 (3)	0.0232 (4)	0.0006 (2)	0.0031 (3)	0.0017 (3)
N1	0.0170 (4)	0.0152 (4)	0.0199 (4)	0.0008 (3)	0.0010 (3)	0.0011 (3)
C1	0.0167 (4)	0.0154 (4)	0.0197 (4)	0.0008 (3)	0.0010 (3)	0.0004 (3)
N2	0.0152 (4)	0.0161 (4)	0.0247 (5)	0.0002 (3)	0.0030 (3)	−0.0001 (3)
C6	0.0152 (4)	0.0139 (4)	0.0213 (4)	0.0010 (3)	0.0010 (3)	0.0006 (3)
N1'	0.0167 (4)	0.0154 (4)	0.0197 (4)	0.0008 (3)	0.0010 (3)	0.0004 (3)
C1'	0.0170 (4)	0.0152 (4)	0.0199 (4)	0.0008 (3)	0.0010 (3)	0.0011 (3)
N2'	0.0152 (4)	0.0139 (4)	0.0213 (4)	0.0010 (3)	0.0010 (3)	0.0006 (3)
C6'	0.0152 (4)	0.0161 (4)	0.0247 (5)	0.0002 (3)	0.0030 (3)	−0.0001 (3)
C2	0.0239 (5)	0.0188 (4)	0.0224 (5)	0.0009 (4)	0.0026 (4)	−0.0029 (4)
C3	0.0283 (6)	0.0269 (6)	0.0205 (5)	0.0035 (5)	0.0034 (4)	−0.0001 (4)
C4	0.0272 (6)	0.0237 (5)	0.0225 (5)	0.0021 (4)	0.0041 (4)	0.0045 (4)
C5	0.0226 (5)	0.0168 (4)	0.0227 (5)	0.0008 (4)	0.0020 (4)	0.0025 (4)
C7	0.0212 (5)	0.0145 (4)	0.0227 (5)	0.0008 (3)	0.0017 (4)	0.0009 (3)
C8	0.0228 (5)	0.0176 (5)	0.0334 (6)	0.0050 (4)	0.0031 (5)	−0.0002 (4)
C9	0.0215 (5)	0.0236 (5)	0.0466 (8)	0.0046 (4)	0.0103 (5)	−0.0014 (5)
C10	0.0187 (5)	0.0209 (5)	0.0390 (7)	−0.0003 (4)	0.0097 (5)	−0.0008 (5)
C11	0.0169 (4)	0.0134 (4)	0.0203 (5)	−0.0006 (3)	0.0023 (3)	0.0006 (3)
C12	0.0189 (4)	0.0148 (4)	0.0225 (5)	−0.0003 (3)	0.0033 (4)	−0.0002 (3)
F1	0.0220 (3)	0.0197 (3)	0.0281 (4)	0.0055 (3)	0.0010 (3)	0.0004 (3)
C13	0.0233 (5)	0.0162 (4)	0.0265 (5)	−0.0019 (4)	0.0072 (4)	−0.0028 (4)
F2	0.0306 (4)	0.0221 (3)	0.0392 (5)	0.0028 (3)	0.0114 (4)	−0.0084 (3)
C14	0.0280 (6)	0.0193 (5)	0.0212 (5)	−0.0071 (4)	0.0061 (4)	−0.0031 (4)
F3	0.0402 (5)	0.0288 (4)	0.0235 (4)	−0.0098 (3)	0.0091 (3)	−0.0074 (3)
C15	0.0243 (5)	0.0183 (4)	0.0194 (5)	−0.0047 (4)	−0.0005 (4)	0.0016 (4)
F4	0.0353 (4)	0.0258 (4)	0.0218 (4)	−0.0030 (3)	−0.0073 (3)	0.0025 (3)
C16	0.0185 (4)	0.0136 (4)	0.0213 (5)	−0.0014 (3)	0.0007 (4)	0.0007 (3)
F5	0.0221 (3)	0.0171 (3)	0.0266 (4)	0.0040 (2)	−0.0036 (3)	0.0004 (3)
C17	0.0155 (4)	0.0165 (4)	0.0233 (5)	0.0000 (3)	0.0033 (4)	−0.0009 (3)
C18	0.0184 (4)	0.0215 (5)	0.0267 (6)	−0.0011 (4)	0.0049 (4)	−0.0008 (4)
F6	0.0240 (4)	0.0300 (4)	0.0250 (4)	0.0003 (3)	0.0054 (3)	0.0031 (3)
C19	0.0229 (5)	0.0334 (7)	0.0362 (7)	−0.0040 (5)	0.0124 (5)	0.0005 (5)
F7	0.0343 (5)	0.0526 (6)	0.0457 (6)	−0.0077 (4)	0.0206 (4)	0.0073 (5)
C20	0.0165 (5)	0.0425 (8)	0.0457 (9)	−0.0052 (5)	0.0086 (5)	−0.0013 (7)
F8	0.0186 (4)	0.0766 (9)	0.0686 (8)	−0.0104 (5)	0.0126 (5)	0.0092 (7)
C21	0.0151 (5)	0.0373 (7)	0.0359 (7)	−0.0003 (5)	0.0012 (5)	0.0014 (6)
F9	0.0162 (4)	0.0625 (7)	0.0466 (6)	−0.0005 (4)	−0.0046 (4)	0.0081 (5)
C22	0.0148 (4)	0.0221 (5)	0.0274 (6)	−0.0001 (4)	0.0022 (4)	−0.0001 (4)
F10	0.0193 (3)	0.0278 (4)	0.0277 (4)	0.0008 (3)	0.0001 (3)	0.0045 (3)
C23	0.039 (2)	0.100 (5)	0.120 (6)	−0.009 (3)	0.026 (3)	−0.029 (4)
C24	0.081 (4)	0.057 (3)	0.095 (5)	−0.023 (3)	0.038 (4)	−0.016 (3)
C25	0.072 (3)	0.054 (3)	0.060 (3)	0.009 (2)	0.032 (3)	0.015 (2)
C26	0.049 (3)	0.060 (3)	0.050 (4)	0.013 (3)	0.021 (3)	0.014 (3)
C27	0.057 (3)	0.070 (3)	0.053 (3)	0.004 (2)	0.015 (2)	−0.018 (2)
O5'	0.0403 (14)	0.0526 (16)	0.0476 (15)	0.0081 (12)	0.0122 (12)	−0.0033 (12)
C23'	0.042 (2)	0.064 (3)	0.050 (2)	−0.0106 (18)	0.0138 (17)	−0.0147 (19)
C24'	0.059 (3)	0.047 (2)	0.075 (3)	0.008 (2)	0.037 (3)	0.001 (2)
C25'	0.071 (3)	0.051 (3)	0.072 (4)	0.003 (2)	0.036 (3)	0.001 (3)
C26'	0.083 (6)	0.053 (3)	0.056 (4)	0.006 (4)	0.026 (4)	0.009 (3)

### Bis[1-oxopyridin-2-olato(1-)]bis(pentafluorophenyl)silicon(IV)–tetrahydrofuran–pentane (2/1/1) (1) Geometric parameters (Å, º)

**Table:**

Si1—O1	1.7910 (9)	C14—F3	1.3353 (15)
Si1—O4	1.8042 (9)	C14—C15	1.3814 (19)
Si1—O3	1.8480 (9)	C15—F4	1.3430 (14)
Si1—O2	1.8503 (9)	C15—C16	1.3853 (17)
Si1—C11	1.9559 (12)	C16—F5	1.3557 (14)
Si1—C17	1.9683 (12)	C17—C18	1.3904 (18)
O1—C1'	1.3324 (14)	C17—C22	1.3957 (17)
O1—N1	1.3324 (14)	C18—F6	1.3543 (15)
O2—N1'	1.3244 (14)	C18—C19	1.3903 (18)
O2—C1	1.3244 (14)	C19—F7	1.3431 (18)
O3—C6'	1.3263 (13)	C19—C20	1.377 (2)
O3—N2	1.3263 (13)	C20—F8	1.3452 (17)
O4—N2'	1.3348 (13)	C20—C21	1.375 (2)
O4—C6	1.3348 (13)	C21—F9	1.3462 (17)
N1—C1	1.3562 (14)	C21—C22	1.3874 (18)
N1—C5	1.3714 (16)	C22—F10	1.3513 (16)
C1—C2	1.3686 (17)	C23—C24	1.434 (10)
N2—C6	1.3487 (15)	C23—H23A	0.9800
N2—C10	1.3706 (16)	C23—H23B	0.9800
C6—C7	1.3740 (15)	C23—H23C	0.9800
N1'—C1'	1.3562 (14)	C24—C25	1.553 (8)
N1'—C2	1.3686 (17)	C24—H24A	0.9900
C1'—C5	1.3714 (16)	C24—H24B	0.9900
N2'—C6'	1.3487 (15)	C25—C26	1.552 (10)
N2'—C7	1.3740 (15)	C25—H25A	0.9900
C6'—C10	1.3706 (16)	C25—H25B	0.9900
C2—C3	1.3732 (19)	C26—C27	1.491 (10)
C2—H2	0.9500	C26—H26A	0.9900
C3—C4	1.4013 (19)	C26—H26B	0.9900
C3—H3	0.9500	C27—H27A	0.9800
C4—C5	1.3828 (19)	C27—H27B	0.9800
C4—H4	0.9500	C27—H27C	0.9800
C5—H5	0.9500	O5'—C23'	1.432 (5)
C7—C8	1.3770 (18)	O5'—C26'	1.456 (10)
C7—H7	0.9500	C23'—C24'	1.498 (6)
C8—C9	1.398 (2)	C23'—H23D	0.9900
C8—H8	0.9500	C23'—H23E	0.9900
C9—C10	1.3776 (19)	C24'—C25'	1.482 (8)
C9—H9	0.9500	C24'—H24C	0.9900
C10—H10	0.9500	C24'—H24D	0.9900
C11—C16	1.3923 (16)	C25'—C26'	1.494 (11)
C11—C12	1.3937 (16)	C25'—H25C	0.9900
C12—F1	1.3545 (14)	C25'—H25D	0.9900
C12—C13	1.3851 (17)	C26'—H26C	0.9900
C13—F2	1.3428 (14)	C26'—H26D	0.9900
C13—C14	1.3861 (19)		
			
O1—Si1—O4	166.74 (4)	C12—C13—C14	119.49 (11)
O1—Si1—O3	86.65 (4)	F3—C14—C15	120.33 (12)
O4—Si1—O3	84.60 (4)	F3—C14—C13	120.99 (12)
O1—Si1—O2	85.17 (4)	C15—C14—C13	118.65 (11)
O4—Si1—O2	84.53 (4)	F4—C15—C14	119.78 (11)
O3—Si1—O2	87.56 (4)	F4—C15—C16	120.51 (11)
O1—Si1—C11	91.41 (4)	C14—C15—C16	119.71 (11)
O4—Si1—C11	98.54 (4)	F5—C16—C15	115.00 (10)
O3—Si1—C11	90.16 (4)	F5—C16—C11	120.69 (10)
O2—Si1—C11	175.99 (5)	C15—C16—C11	124.29 (11)
O1—Si1—C17	99.44 (5)	C18—C17—C22	113.39 (11)
O4—Si1—C17	88.68 (4)	C18—C17—Si1	122.91 (9)
O3—Si1—C17	172.66 (4)	C22—C17—Si1	123.45 (9)
O2—Si1—C17	88.88 (5)	F6—C18—C19	115.25 (12)
C11—Si1—C17	93.75 (5)	F6—C18—C17	120.49 (11)
C1'—O1—Si1	112.84 (7)	C19—C18—C17	124.25 (12)
N1—O1—Si1	112.84 (7)	F7—C19—C20	119.76 (13)
N1'—O2—Si1	111.23 (7)	F7—C19—C18	120.96 (14)
C1—O2—Si1	111.23 (7)	C20—C19—C18	119.28 (13)
C6'—O3—Si1	110.81 (7)	F8—C20—C21	120.52 (15)
N2—O3—Si1	110.81 (7)	F8—C20—C19	120.04 (15)
N2'—O4—Si1	111.71 (7)	C21—C20—C19	119.44 (13)
C6—O4—Si1	111.71 (7)	F9—C21—C20	119.99 (13)
O1—N1—C1	114.69 (9)	F9—C21—C22	120.74 (14)
O1—N1—C5	124.26 (10)	C20—C21—C22	119.27 (13)
C1—N1—C5	121.02 (11)	F10—C22—C21	114.91 (11)
O2—C1—N1	114.61 (10)	F10—C22—C17	120.77 (10)
O2—C1—C2	123.66 (10)	C21—C22—C17	124.32 (13)
N1—C1—C2	121.70 (10)	C24—C23—H23A	109.5
O3—N2—C6	114.78 (9)	C24—C23—H23B	109.5
O3—N2—C10	124.02 (10)	H23A—C23—H23B	109.5
C6—N2—C10	121.20 (10)	C24—C23—H23C	109.5
O4—C6—N2	114.45 (9)	H23A—C23—H23C	109.5
O4—C6—C7	124.04 (10)	H23B—C23—H23C	109.5
N2—C6—C7	121.50 (10)	C23—C24—C25	115.3 (6)
O2—N1'—C1'	114.61 (10)	C23—C24—H24A	108.4
O2—N1'—C2	123.66 (10)	C25—C24—H24A	108.4
C1'—N1'—C2	121.70 (10)	C23—C24—H24B	108.4
O1—C1'—N1'	114.69 (9)	C25—C24—H24B	108.4
O1—C1'—C5	124.26 (10)	H24A—C24—H24B	107.5
N1'—C1'—C5	121.02 (11)	C26—C25—C24	109.8 (6)
O4—N2'—C6'	114.45 (9)	C26—C25—H25A	109.7
O4—N2'—C7	124.04 (10)	C24—C25—H25A	109.7
C6'—N2'—C7	121.50 (10)	C26—C25—H25B	109.7
O3—C6'—N2'	114.78 (9)	C24—C25—H25B	109.7
O3—C6'—C10	124.02 (10)	H25A—C25—H25B	108.2
N2'—C6'—C10	121.20 (10)	C27—C26—C25	114.5 (6)
N1'—C2—C3	118.72 (11)	C27—C26—H26A	108.6
C1—C2—C3	118.72 (11)	C25—C26—H26A	108.6
C1—C2—H2	120.6	C27—C26—H26B	108.6
C3—C2—H2	120.6	C25—C26—H26B	108.6
C2—C3—C4	119.88 (12)	H26A—C26—H26B	107.6
C2—C3—H3	120.1	C26—C27—H27A	109.5
C4—C3—H3	120.1	C26—C27—H27B	109.5
C5—C4—C3	120.26 (12)	H27A—C27—H27B	109.5
C5—C4—H4	119.9	C26—C27—H27C	109.5
C3—C4—H4	119.9	H27A—C27—H27C	109.5
C1'—C5—C4	118.42 (11)	H27B—C27—H27C	109.5
N1—C5—C4	118.42 (11)	C23'—O5'—C26'	109.5 (5)
N1—C5—H5	120.8	O5'—C23'—C24'	104.9 (4)
C4—C5—H5	120.8	O5'—C23'—H23D	110.8
N2'—C7—C8	118.54 (11)	C24'—C23'—H23D	110.8
C6—C7—C8	118.54 (11)	O5'—C23'—H23E	110.8
C6—C7—H7	120.7	C24'—C23'—H23E	110.8
C8—C7—H7	120.7	H23D—C23'—H23E	108.8
C7—C8—C9	119.84 (11)	C25'—C24'—C23'	102.3 (4)
C7—C8—H8	120.1	C25'—C24'—H24C	111.3
C9—C8—H8	120.1	C23'—C24'—H24C	111.3
C10—C9—C8	120.24 (12)	C25'—C24'—H24D	111.3
C10—C9—H9	119.9	C23'—C24'—H24D	111.3
C8—C9—H9	119.9	H24C—C24'—H24D	109.2
C6'—C10—C9	118.59 (12)	C24'—C25'—C26'	105.0 (6)
N2—C10—C9	118.59 (12)	C24'—C25'—H25C	110.7
N2—C10—H10	120.7	C26'—C25'—H25C	110.7
C9—C10—H10	120.7	C24'—C25'—H25D	110.7
C16—C11—C12	113.42 (10)	C26'—C25'—H25D	110.7
C16—C11—Si1	123.97 (8)	H25C—C25'—H25D	108.8
C12—C11—Si1	122.43 (8)	O5'—C26'—C25'	104.9 (7)
F1—C12—C13	115.50 (10)	O5'—C26'—H26C	110.8
F1—C12—C11	120.11 (10)	C25'—C26'—H26C	110.8
C13—C12—C11	124.37 (11)	O5'—C26'—H26D	110.8
F2—C13—C12	120.66 (12)	C25'—C26'—H26D	110.8
F2—C13—C14	119.84 (11)	H26C—C26'—H26D	108.8
			
O4—Si1—O1—C1'	−28.5 (2)	O1—C1'—C5—C4	178.47 (11)
O3—Si1—O1—C1'	−77.23 (8)	N1'—C1'—C5—C4	0.59 (18)
O2—Si1—O1—C1'	10.60 (8)	O1—N1—C5—C4	178.47 (11)
C11—Si1—O1—C1'	−167.31 (8)	C1—N1—C5—C4	0.59 (18)
C17—Si1—O1—C1'	98.65 (8)	C3—C4—C5—C1'	−0.66 (19)
O4—Si1—O1—N1	−28.5 (2)	C3—C4—C5—N1	−0.66 (19)
O3—Si1—O1—N1	−77.23 (8)	O4—N2'—C7—C8	176.47 (11)
O2—Si1—O1—N1	10.60 (8)	C6'—N2'—C7—C8	−3.09 (18)
C11—Si1—O1—N1	−167.31 (8)	O4—C6—C7—C8	176.47 (11)
C17—Si1—O1—N1	98.65 (8)	N2—C6—C7—C8	−3.09 (18)
O1—Si1—O2—N1'	−10.44 (7)	N2'—C7—C8—C9	0.8 (2)
O4—Si1—O2—N1'	161.20 (8)	C6—C7—C8—C9	0.8 (2)
O3—Si1—O2—N1'	76.40 (7)	C7—C8—C9—C10	1.3 (2)
C17—Si1—O2—N1'	−110.02 (8)	O3—C6'—C10—C9	179.41 (13)
O1—Si1—O2—C1	−10.44 (7)	N2'—C6'—C10—C9	−1.0 (2)
O4—Si1—O2—C1	161.20 (8)	O3—N2—C10—C9	179.41 (13)
O3—Si1—O2—C1	76.40 (7)	C6—N2—C10—C9	−1.0 (2)
C17—Si1—O2—C1	−110.02 (8)	C8—C9—C10—C6'	−1.3 (2)
O1—Si1—O3—C6'	154.54 (8)	C8—C9—C10—N2	−1.3 (2)
O4—Si1—O3—C6'	−15.49 (8)	C16—C11—C12—F1	179.86 (10)
O2—Si1—O3—C6'	69.23 (8)	Si1—C11—C12—F1	4.45 (15)
C11—Si1—O3—C6'	−114.06 (8)	C16—C11—C12—C13	1.43 (17)
O1—Si1—O3—N2	154.54 (8)	Si1—C11—C12—C13	−173.97 (9)
O4—Si1—O3—N2	−15.49 (8)	F1—C12—C13—F2	1.51 (17)
O2—Si1—O3—N2	69.23 (8)	C11—C12—C13—F2	180.00 (11)
C11—Si1—O3—N2	−114.06 (8)	F1—C12—C13—C14	−177.48 (11)
O1—Si1—O4—N2'	−31.6 (2)	C11—C12—C13—C14	1.01 (19)
O3—Si1—O4—N2'	17.32 (7)	F2—C13—C14—F3	−3.34 (18)
O2—Si1—O4—N2'	−70.74 (8)	C12—C13—C14—F3	175.65 (11)
C11—Si1—O4—N2'	106.66 (8)	F2—C13—C14—C15	178.54 (11)
C17—Si1—O4—N2'	−159.74 (8)	C12—C13—C14—C15	−2.46 (18)
O1—Si1—O4—C6	−31.6 (2)	F3—C14—C15—F4	2.55 (18)
O3—Si1—O4—C6	17.32 (7)	C13—C14—C15—F4	−179.32 (11)
O2—Si1—O4—C6	−70.74 (8)	F3—C14—C15—C16	−176.71 (11)
C11—Si1—O4—C6	106.66 (8)	C13—C14—C15—C16	1.41 (18)
C17—Si1—O4—C6	−159.74 (8)	F4—C15—C16—F5	0.51 (16)
Si1—O1—N1—C1	−8.78 (12)	C14—C15—C16—F5	179.77 (11)
Si1—O1—N1—C5	173.22 (9)	F4—C15—C16—C11	−178.03 (11)
Si1—O2—C1—N1	8.22 (12)	C14—C15—C16—C11	1.23 (19)
Si1—O2—C1—C2	−173.65 (9)	C12—C11—C16—F5	178.98 (10)
O1—N1—C1—O2	0.12 (14)	Si1—C11—C16—F5	−5.70 (16)
C5—N1—C1—O2	178.19 (10)	C12—C11—C16—C15	−2.56 (17)
O1—N1—C1—C2	−178.04 (10)	Si1—C11—C16—C15	172.76 (9)
C5—N1—C1—C2	0.02 (17)	C22—C17—C18—F6	178.41 (11)
Si1—O3—N2—C6	10.62 (12)	Si1—C17—C18—F6	−7.13 (16)
Si1—O3—N2—C10	−169.72 (11)	C22—C17—C18—C19	−2.95 (19)
Si1—O4—C6—N2	−15.96 (12)	Si1—C17—C18—C19	171.50 (11)
Si1—O4—C6—C7	164.44 (9)	F6—C18—C19—F7	1.0 (2)
O3—N2—C6—O4	3.27 (15)	C17—C18—C19—F7	−177.71 (13)
C10—N2—C6—O4	−176.40 (11)	F6—C18—C19—C20	−179.78 (14)
O3—N2—C6—C7	−177.13 (11)	C17—C18—C19—C20	1.5 (2)
C10—N2—C6—C7	3.20 (18)	F7—C19—C20—F8	−0.9 (3)
Si1—O2—N1'—C1'	8.22 (12)	C18—C19—C20—F8	179.89 (15)
Si1—O2—N1'—C2	−173.65 (9)	F7—C19—C20—C21	179.80 (15)
Si1—O1—C1'—N1'	−8.78 (12)	C18—C19—C20—C21	0.6 (2)
Si1—O1—C1'—C5	173.22 (9)	F8—C20—C21—F9	0.5 (3)
O2—N1'—C1'—O1	0.12 (14)	C19—C20—C21—F9	179.87 (15)
C2—N1'—C1'—O1	−178.04 (10)	F8—C20—C21—C22	179.77 (15)
O2—N1'—C1'—C5	178.19 (10)	C19—C20—C21—C22	−0.9 (3)
C2—N1'—C1'—C5	0.02 (17)	F9—C21—C22—F10	−0.7 (2)
Si1—O4—N2'—C6'	−15.96 (12)	C20—C21—C22—F10	−179.93 (14)
Si1—O4—N2'—C7	164.44 (9)	F9—C21—C22—C17	178.44 (13)
Si1—O3—C6'—N2'	10.62 (12)	C20—C21—C22—C17	−0.8 (2)
Si1—O3—C6'—C10	−169.72 (11)	C18—C17—C22—F10	−178.32 (11)
O4—N2'—C6'—O3	3.27 (15)	Si1—C17—C22—F10	7.26 (17)
C7—N2'—C6'—O3	−177.13 (11)	C18—C17—C22—C21	2.58 (19)
O4—N2'—C6'—C10	−176.40 (11)	Si1—C17—C22—C21	−171.84 (11)
C7—N2'—C6'—C10	3.20 (18)	C23—C24—C25—C26	−72.1 (8)
O2—N1'—C2—C3	−178.57 (11)	C24—C25—C26—C27	173.7 (6)
C1'—N1'—C2—C3	−0.58 (18)	C26'—O5'—C23'—C24'	−20.7 (6)
O2—C1—C2—C3	−178.57 (11)	O5'—C23'—C24'—C25'	34.3 (6)
N1—C1—C2—C3	−0.58 (18)	C23'—C24'—C25'—C26'	−35.2 (7)
N1'—C2—C3—C4	0.5 (2)	C23'—O5'—C26'—C25'	−1.3 (7)
C1—C2—C3—C4	0.5 (2)	C24'—C25'—C26'—O5'	23.2 (8)
C2—C3—C4—C5	0.1 (2)		

### Bis[1-oxopyridin-2-olato(1-)]bis(pentafluorophenyl)silicon(IV)–tetrahydrofuran–pentane (2/1/1) (1) Hydrogen-bond geometry (Å, º)

**Table:**

*D*—H···*A*	*D*—H	H···*A*	*D*···*A*	*D*—H···*A*
C2—H2···F1^i^	0.95	2.34	3.2809 (15)	170
C3—H3···F7^ii^	0.95	3.23	3.6665 (18)	110
C4—H4···F6^iii^	0.95	2.68	3.5757 (16)	158
C5—H5···F4^iv^	0.95	2.59	3.2997 (15)	132
C7—H7···F5^v^	0.95	2.57	3.2307 (14)	127
C8—H8···F5^v^	0.95	2.77	3.3319 (16)	119
C8—H8···F6^v^	0.95	2.56	3.2230 (15)	127
C8—H8···F9^i^	0.95	2.81	3.2507 (18)	110
C9—H9···F6^v^	0.95	3.31	3.6095 (17)	101
C9—H9···F8^vi^	0.95	2.80	3.2885 (18)	113
C10—H10···F2^iv^	0.95	2.73	3.3522 (16)	124
C10—H10···F8^vi^	0.95	2.37	3.0797 (16)	131

Symmetry codes: (i) −*x*+3/2, *y*+1/2, −*z*+3/2; (ii) *x*−1/2, −*y*+3/2, *z*+1/2; (iii) −*x*+3/2, *y*−1/2, −*z*+3/2; (iv) −*x*+1, −*y*+1, −*z*+1; (v) −*x*+1, −*y*+2, −*z*+1; (vi) *x*−1, *y*, *z*.

### Bis[1-oxopyridin-2-olato(1-)]bis(4-mwthylphenyl)silicon(IV) (2) Crystal data

**Table:**

C_24_H_22_N_2_O_4_Si	*Z* = 2
*M**_r_* = 430.52	*F*(000) = 452
Triclinic, *P*1	*D*_x_ = 1.398 Mg m^−^^3^
*a* = 8.5662 (8) Å	Mo *K*α radiation, λ = 0.71073 Å
*b* = 8.8343 (8) Å	Cell parameters from 4074 reflections
*c* = 14.7801 (14) Å	θ = 2.4–30.3°
α = 93.057 (2)°	µ = 0.15 mm^−^^1^
β = 105.3716 (19)°	*T* = 100 K
γ = 106.7565 (18)°	Block, colorless
*V* = 1022.45 (17) Å^3^	0.24 × 0.24 × 0.20 mm

### Bis[1-oxopyridin-2-olato(1-)]bis(4-mwthylphenyl)silicon(IV) (2) Data collection

**Table:**

Bruker SMART APEXII CCD platform diffractometer	4231 reflections with *I* > 2σ(*I*)
Radiation source: fine-focus sealed tube	*R*_int_ = 0.065
ω scans	θ_max_ = 30.6°, θ_min_ = 2.4°
Absorption correction: multi-scan (SADABS; Krause *et al.*, 2015)	*h* = −12→12
*T*_min_ = 0.695, *T*_max_ = 0.746	*k* = −12→12
25883 measured reflections	*l* = −21→21
6239 independent reflections	

### Bis[1-oxopyridin-2-olato(1-)]bis(4-mwthylphenyl)silicon(IV) (2) Refinement

**Table:**

Refinement on *F*^2^	Primary atom site location: dual
Least-squares matrix: full	Secondary atom site location: difference Fourier map
*R*[*F*^2^ > 2σ(*F*^2^)] = 0.054	Hydrogen site location: inferred from neighbouring sites
*wR*(*F*^2^) = 0.149	H-atom parameters constrained
*S* = 1.05	*w* = 1/[σ^2^(*F*_o_^2^) + (0.061*P*)^2^ + 0.4958*P*] where *P* = (*F*_o_^2^ + 2*F*_c_^2^)/3
6239 reflections	(Δ/σ)_max_ = 0.001
284 parameters	Δρ_max_ = 1.01 e Å^−^^3^
0 restraints	Δρ_min_ = −0.43 e Å^−^^3^

### Bis[1-oxopyridin-2-olato(1-)]bis(4-mwthylphenyl)silicon(IV) (2) Special details

**Table:**

Geometry. All esds (except the esd in the dihedral angle between two l.s. planes) are estimated using the full covariance matrix. The cell esds are taken into account individually in the estimation of esds in distances, angles and torsion angles; correlations between esds in cell parameters are only used when they are defined by crystal symmetry. An approximate (isotropic) treatment of cell esds is used for estimating esds involving l.s. planes.
Refinement. Both bidentate ligands are disordered with the coplanar flips of themselves (0.658 (19):0.342 (19) and 0.612 (19):0.388 (19) for the rings containing C1/N1 and C6/N2, respectively). Due to resolution limitations, the disorder model did not include the entire ring, but was modeled by refining the occupancies of the two atoms types (C and N) at the oxygen-coordinating portions of the rings. The occupancies at each site were constrained to sum to one and additionally to sum to one C and one N atom between the two sites on each ring. The positional and anisotropic displacement parameters, espectively, at each site of disorder were constrained to be equivalent. It is understood that this type of disorder model will likely exhibit a weighted average of Si–O bond lengths, trending with the disorder ratios.

### Bis[1-oxopyridin-2-olato(1-)]bis(4-mwthylphenyl)silicon(IV) (2) Fractional atomic coordinates and isotropic or equivalent isotropic displacement parameters (Å^2^)

**Table:**

	*x*	*y*	*z*	*U*_iso_*/*U*_eq_	Occ. (<1)
Si1	0.46085 (7)	0.32324 (6)	0.72662 (4)	0.01840 (13)	
O1	0.62723 (17)	0.50446 (16)	0.78902 (10)	0.0207 (3)	
O2	0.64598 (17)	0.23371 (16)	0.75110 (10)	0.0219 (3)	
O3	0.33449 (17)	0.13549 (16)	0.65212 (9)	0.0202 (3)	
O4	0.53005 (18)	0.37788 (16)	0.61623 (10)	0.0225 (3)	
N1	0.7819 (2)	0.4865 (2)	0.82216 (12)	0.0200 (4)	0.658 (19)
C1	0.7904 (2)	0.3388 (2)	0.80021 (13)	0.0210 (4)	0.658 (19)
N2	0.3498 (2)	0.1276 (2)	0.56448 (12)	0.0198 (4)	0.612 (19)
C6	0.4563 (2)	0.2595 (2)	0.54532 (13)	0.0211 (4)	0.612 (19)
N1'	0.7904 (2)	0.3388 (2)	0.80021 (13)	0.0210 (4)	0.342 (19)
C1'	0.7819 (2)	0.4865 (2)	0.82216 (12)	0.0200 (4)	0.342 (19)
N2'	0.4563 (2)	0.2595 (2)	0.54532 (13)	0.0211 (4)	0.388 (19)
C6'	0.3498 (2)	0.1276 (2)	0.56448 (12)	0.0198 (4)	0.388 (19)
C2	0.9459 (3)	0.3113 (3)	0.83015 (14)	0.0241 (4)	
H2	0.954380	0.208720	0.814746	0.029*	
C3	1.0883 (3)	0.4329 (3)	0.88228 (15)	0.0269 (4)	
H3	1.195403	0.414653	0.902786	0.032*	
C4	1.0749 (3)	0.5832 (3)	0.90496 (15)	0.0283 (5)	
H4	1.172679	0.667323	0.941344	0.034*	
C5	0.9201 (3)	0.6090 (3)	0.87452 (15)	0.0248 (4)	
H5	0.909370	0.710641	0.889705	0.030*	
C7	0.4840 (3)	0.2623 (3)	0.45720 (14)	0.0248 (4)	
H7	0.558326	0.354501	0.443053	0.030*	
C8	0.4013 (3)	0.1286 (3)	0.39071 (15)	0.0282 (5)	
H8	0.418987	0.128457	0.329907	0.034*	
C9	0.2919 (3)	−0.0068 (3)	0.41123 (15)	0.0279 (5)	
H9	0.235815	−0.098844	0.364838	0.034*	
C10	0.2658 (3)	−0.0062 (2)	0.49899 (14)	0.0242 (4)	
H10	0.190706	−0.097031	0.513907	0.029*	
C11	0.2970 (2)	0.4356 (2)	0.69348 (13)	0.0176 (4)	
C12	0.3356 (3)	0.5987 (2)	0.72212 (14)	0.0232 (4)	
H12	0.448901	0.656954	0.758615	0.028*	
C13	0.2159 (3)	0.6806 (3)	0.69979 (15)	0.0252 (4)	
H13	0.249201	0.791922	0.720466	0.030*	
C14	0.0474 (3)	0.5988 (3)	0.64712 (15)	0.0256 (4)	
C15	0.0053 (3)	0.4365 (3)	0.61821 (16)	0.0280 (5)	
H15	−0.108304	0.378531	0.582028	0.034*	
C16	0.1265 (3)	0.3562 (3)	0.64118 (15)	0.0255 (4)	
H16	0.092432	0.244689	0.620812	0.031*	
C17	−0.0844 (3)	0.6841 (3)	0.62348 (18)	0.0337 (5)	
H17A	−0.124820	0.680593	0.554615	0.051*	
H17B	−0.033391	0.795498	0.653631	0.051*	
H17C	−0.180549	0.631563	0.646813	0.051*	
C18	0.4015 (2)	0.2402 (2)	0.83540 (13)	0.0176 (4)	
C19	0.2635 (3)	0.1029 (2)	0.82682 (15)	0.0261 (4)	
H19	0.195061	0.050060	0.765313	0.031*	
C20	0.2234 (3)	0.0412 (3)	0.90568 (16)	0.0303 (5)	
H20	0.128409	−0.052066	0.896705	0.036*	
C21	0.3202 (3)	0.1141 (3)	0.99752 (15)	0.0266 (4)	
C22	0.4531 (3)	0.2532 (3)	1.00683 (15)	0.0275 (4)	
H22	0.518803	0.308042	1.068349	0.033*	
C23	0.4923 (3)	0.3146 (3)	0.92768 (15)	0.0255 (4)	
H23	0.584245	0.410556	0.936991	0.031*	
C24	0.2834 (3)	0.0407 (3)	1.08269 (17)	0.0371 (6)	
H24A	0.163999	−0.026642	1.065461	0.056*	
H24B	0.304562	0.125643	1.134065	0.056*	
H24C	0.357891	−0.024422	1.103770	0.056*	

### Bis[1-oxopyridin-2-olato(1-)]bis(4-mwthylphenyl)silicon(IV) (2) Atomic displacement parameters (Å^2^)

**Table:**

	*U*^11^	*U*^22^	*U*^33^	*U*^12^	*U*^13^	*U*^23^
Si1	0.0199 (3)	0.0163 (3)	0.0186 (3)	0.00566 (19)	0.0056 (2)	0.00027 (19)
O1	0.0170 (6)	0.0180 (7)	0.0257 (7)	0.0070 (5)	0.0028 (5)	−0.0002 (5)
O2	0.0197 (7)	0.0190 (7)	0.0274 (7)	0.0076 (5)	0.0068 (6)	−0.0006 (5)
O3	0.0252 (7)	0.0200 (7)	0.0156 (6)	0.0062 (5)	0.0078 (5)	−0.0001 (5)
O4	0.0261 (7)	0.0206 (7)	0.0214 (7)	0.0058 (6)	0.0100 (6)	0.0003 (5)
N1	0.0170 (8)	0.0218 (9)	0.0224 (9)	0.0065 (7)	0.0069 (7)	0.0041 (7)
C1	0.0227 (9)	0.0231 (9)	0.0210 (9)	0.0097 (7)	0.0096 (7)	0.0051 (7)
N2	0.0224 (9)	0.0228 (9)	0.0167 (8)	0.0121 (7)	0.0049 (7)	0.0007 (6)
C6	0.0263 (10)	0.0222 (9)	0.0192 (9)	0.0126 (8)	0.0082 (7)	0.0039 (7)
N1'	0.0227 (9)	0.0231 (9)	0.0210 (9)	0.0097 (7)	0.0096 (7)	0.0051 (7)
C1'	0.0170 (8)	0.0218 (9)	0.0224 (9)	0.0065 (7)	0.0069 (7)	0.0041 (7)
N2'	0.0263 (10)	0.0222 (9)	0.0192 (9)	0.0126 (8)	0.0082 (7)	0.0039 (7)
C6'	0.0224 (9)	0.0228 (9)	0.0167 (8)	0.0121 (7)	0.0049 (7)	0.0007 (6)
C2	0.0265 (10)	0.0287 (11)	0.0232 (10)	0.0142 (8)	0.0110 (8)	0.0058 (8)
C3	0.0208 (10)	0.0371 (12)	0.0285 (11)	0.0144 (9)	0.0101 (8)	0.0092 (9)
C4	0.0174 (9)	0.0336 (12)	0.0286 (11)	0.0032 (8)	0.0036 (8)	0.0040 (9)
C5	0.0213 (10)	0.0219 (10)	0.0304 (11)	0.0055 (8)	0.0074 (8)	0.0045 (8)
C7	0.0297 (11)	0.0307 (11)	0.0208 (9)	0.0167 (9)	0.0100 (8)	0.0074 (8)
C8	0.0335 (12)	0.0360 (12)	0.0205 (10)	0.0194 (10)	0.0082 (9)	0.0014 (8)
C9	0.0303 (11)	0.0290 (11)	0.0230 (10)	0.0117 (9)	0.0044 (8)	−0.0044 (8)
C10	0.0249 (10)	0.0224 (10)	0.0249 (10)	0.0104 (8)	0.0042 (8)	−0.0021 (8)
C11	0.0187 (9)	0.0202 (9)	0.0158 (8)	0.0071 (7)	0.0068 (7)	0.0037 (7)
C12	0.0210 (9)	0.0243 (10)	0.0236 (10)	0.0074 (8)	0.0052 (8)	0.0023 (8)
C13	0.0261 (10)	0.0230 (10)	0.0286 (11)	0.0101 (8)	0.0090 (8)	0.0045 (8)
C14	0.0239 (10)	0.0301 (11)	0.0271 (10)	0.0131 (9)	0.0082 (8)	0.0106 (8)
C15	0.0218 (10)	0.0271 (11)	0.0301 (11)	0.0045 (8)	0.0031 (8)	0.0044 (8)
C16	0.0214 (10)	0.0242 (10)	0.0272 (10)	0.0041 (8)	0.0048 (8)	0.0019 (8)
C17	0.0275 (11)	0.0358 (13)	0.0419 (13)	0.0156 (10)	0.0090 (10)	0.0146 (10)
C18	0.0191 (9)	0.0148 (8)	0.0218 (9)	0.0079 (7)	0.0082 (7)	0.0021 (7)
C19	0.0327 (11)	0.0190 (10)	0.0236 (10)	0.0050 (8)	0.0074 (8)	0.0001 (8)
C20	0.0385 (12)	0.0195 (10)	0.0298 (11)	0.0016 (9)	0.0131 (10)	0.0041 (8)
C21	0.0346 (12)	0.0251 (10)	0.0276 (10)	0.0147 (9)	0.0141 (9)	0.0103 (8)
C22	0.0271 (11)	0.0342 (12)	0.0200 (10)	0.0100 (9)	0.0050 (8)	0.0025 (8)
C23	0.0232 (10)	0.0275 (11)	0.0234 (10)	0.0045 (8)	0.0075 (8)	0.0008 (8)
C24	0.0485 (15)	0.0360 (13)	0.0327 (12)	0.0147 (11)	0.0184 (11)	0.0140 (10)

### Bis[1-oxopyridin-2-olato(1-)]bis(4-mwthylphenyl)silicon(IV) (2) Geometric parameters (Å, º)

**Table:**

Si1—O3	1.8093 (14)	C7—H7	0.9500
Si1—O1	1.8097 (14)	C8—C9	1.395 (3)
Si1—O4	1.9179 (15)	C8—H8	0.9500
Si1—C11	1.9202 (19)	C9—C10	1.373 (3)
Si1—O2	1.9290 (15)	C9—H9	0.9500
Si1—C18	1.9301 (19)	C10—H10	0.9500
O1—C1'	1.344 (2)	C11—C12	1.396 (3)
O1—N1	1.344 (2)	C11—C16	1.406 (3)
O2—N1'	1.307 (2)	C12—C13	1.399 (3)
O2—C1	1.307 (2)	C12—H12	0.9500
O3—C6'	1.336 (2)	C13—C14	1.398 (3)
O3—N2	1.336 (2)	C13—H13	0.9500
O4—N2'	1.320 (2)	C14—C15	1.387 (3)
O4—C6	1.320 (2)	C14—C17	1.508 (3)
N1—C1	1.356 (3)	C15—C16	1.400 (3)
N1—C5	1.363 (3)	C15—H15	0.9500
C1—C2	1.384 (3)	C16—H16	0.9500
N2—C6	1.353 (3)	C17—H17A	0.9800
N2—C10	1.367 (3)	C17—H17B	0.9800
C6—C7	1.384 (3)	C17—H17C	0.9800
N1'—C1'	1.356 (3)	C18—C23	1.395 (3)
N1'—C2	1.384 (3)	C18—C19	1.401 (3)
C1'—C5	1.363 (3)	C19—C20	1.395 (3)
N2'—C6'	1.353 (3)	C19—H19	0.9500
N2'—C7	1.384 (3)	C20—C21	1.395 (3)
C6'—C10	1.367 (3)	C20—H20	0.9500
C2—C3	1.376 (3)	C21—C22	1.385 (3)
C2—H2	0.9500	C21—C24	1.511 (3)
C3—C4	1.396 (3)	C22—C23	1.395 (3)
C3—H3	0.9500	C22—H22	0.9500
C4—C5	1.372 (3)	C23—H23	0.9500
C4—H4	0.9500	C24—H24A	0.9800
C5—H5	0.9500	C24—H24B	0.9800
C7—C8	1.374 (3)	C24—H24C	0.9800
			
O3—Si1—O1	165.96 (7)	C8—C7—N2'	118.4 (2)
O3—Si1—O4	83.76 (6)	C8—C7—H7	120.8
O1—Si1—O4	86.24 (7)	C6—C7—H7	120.8
O3—Si1—C11	98.02 (8)	C7—C8—C9	120.9 (2)
O1—Si1—C11	91.68 (7)	C7—C8—H8	119.5
O4—Si1—C11	89.37 (7)	C9—C8—H8	119.5
O3—Si1—O2	85.72 (6)	C10—C9—C8	119.52 (19)
O1—Si1—O2	83.35 (6)	C10—C9—H9	120.2
O4—Si1—O2	83.28 (6)	C8—C9—H9	120.2
C11—Si1—O2	171.36 (8)	C6'—C10—C9	118.8 (2)
O3—Si1—C18	91.04 (7)	N2—C10—C9	118.8 (2)
O1—Si1—C18	97.64 (8)	N2—C10—H10	120.6
O4—Si1—C18	171.40 (7)	C9—C10—H10	120.6
C11—Si1—C18	98.16 (8)	C12—C11—C16	115.36 (18)
O2—Si1—C18	89.53 (7)	C12—C11—Si1	123.04 (15)
C1'—O1—Si1	114.21 (11)	C16—C11—Si1	121.55 (15)
N1—O1—Si1	114.21 (11)	C11—C12—C13	123.31 (19)
N1'—O2—Si1	111.83 (12)	C11—C12—H12	118.3
C1—O2—Si1	111.83 (12)	C13—C12—H12	118.3
C6'—O3—Si1	114.16 (12)	C14—C13—C12	120.1 (2)
N2—O3—Si1	114.16 (12)	C14—C13—H13	119.9
N2'—O4—Si1	111.43 (12)	C12—C13—H13	119.9
C6—O4—Si1	111.43 (12)	C15—C14—C13	117.77 (19)
O1—N1—C1	115.44 (16)	C15—C14—C17	121.2 (2)
O1—N1—C5	121.98 (17)	C13—C14—C17	121.0 (2)
C1—N1—C5	122.57 (17)	C14—C15—C16	121.4 (2)
O2—C1—N1	115.09 (16)	C14—C15—H15	119.3
O2—C1—C2	125.89 (18)	C16—C15—H15	119.3
N1—C1—C2	119.02 (18)	C15—C16—C11	122.0 (2)
O3—N2—C6	115.62 (16)	C15—C16—H16	119.0
O3—N2—C10	122.21 (18)	C11—C16—H16	119.0
C6—N2—C10	122.16 (17)	C14—C17—H17A	109.5
O4—C6—N2	115.03 (16)	C14—C17—H17B	109.5
O4—C6—C7	124.77 (18)	H17A—C17—H17B	109.5
N2—C6—C7	120.18 (18)	C14—C17—H17C	109.5
O2—N1'—C1'	115.09 (16)	H17A—C17—H17C	109.5
O2—N1'—C2	125.89 (18)	H17B—C17—H17C	109.5
C1'—N1'—C2	119.02 (18)	C23—C18—C19	115.71 (18)
O1—C1'—N1'	115.44 (16)	C23—C18—Si1	121.98 (15)
O1—C1'—C5	121.98 (17)	C19—C18—Si1	122.31 (15)
N1'—C1'—C5	122.57 (17)	C20—C19—C18	122.1 (2)
O4—N2'—C6'	115.03 (16)	C20—C19—H19	118.9
O4—N2'—C7	124.77 (18)	C18—C19—H19	118.9
C6'—N2'—C7	120.18 (18)	C19—C20—C21	121.1 (2)
O3—C6'—N2'	115.62 (16)	C19—C20—H20	119.4
O3—C6'—C10	122.21 (18)	C21—C20—H20	119.4
N2'—C6'—C10	122.16 (17)	C22—C21—C20	117.22 (19)
C3—C2—C1	119.8 (2)	C22—C21—C24	121.8 (2)
C3—C2—N1'	119.8 (2)	C20—C21—C24	120.9 (2)
C3—C2—H2	120.1	C21—C22—C23	121.3 (2)
C1—C2—H2	120.1	C21—C22—H22	119.3
C2—C3—C4	119.81 (19)	C23—C22—H22	119.3
C2—C3—H3	120.1	C18—C23—C22	122.4 (2)
C4—C3—H3	120.1	C18—C23—H23	118.8
C5—C4—C3	119.8 (2)	C22—C23—H23	118.8
C5—C4—H4	120.1	C21—C24—H24A	109.5
C3—C4—H4	120.1	C21—C24—H24B	109.5
C1'—C5—C4	119.0 (2)	H24A—C24—H24B	109.5
N1—C5—C4	119.0 (2)	C21—C24—H24C	109.5
N1—C5—H5	120.5	H24A—C24—H24C	109.5
C4—C5—H5	120.5	H24B—C24—H24C	109.5
C8—C7—C6	118.4 (2)		
			
O3—Si1—O1—C1'	−41.7 (3)	C7—N2'—C6'—O3	178.59 (16)
O4—Si1—O1—C1'	−86.26 (13)	O4—N2'—C6'—C10	−178.92 (17)
C11—Si1—O1—C1'	−175.52 (13)	C7—N2'—C6'—C10	−0.2 (3)
O2—Si1—O1—C1'	−2.61 (12)	O2—C1—C2—C3	−179.47 (18)
C18—Si1—O1—C1'	86.02 (13)	N1—C1—C2—C3	0.7 (3)
O3—Si1—O1—N1	−41.7 (3)	O2—N1'—C2—C3	−179.47 (18)
O4—Si1—O1—N1	−86.26 (13)	C1'—N1'—C2—C3	0.7 (3)
C11—Si1—O1—N1	−175.52 (13)	C1—C2—C3—C4	0.2 (3)
O2—Si1—O1—N1	−2.61 (12)	N1'—C2—C3—C4	0.2 (3)
C18—Si1—O1—N1	86.02 (13)	C2—C3—C4—C5	−0.5 (3)
O1—Si1—O3—C6'	−44.8 (3)	O1—C1'—C5—C4	−179.03 (18)
O4—Si1—O3—C6'	−0.04 (12)	N1'—C1'—C5—C4	1.2 (3)
C11—Si1—O3—C6'	88.43 (13)	O1—N1—C5—C4	−179.03 (18)
O2—Si1—O3—C6'	−83.73 (12)	C1—N1—C5—C4	1.2 (3)
C18—Si1—O3—C6'	−173.18 (13)	C3—C4—C5—C1'	−0.2 (3)
O1—Si1—O3—N2	−44.8 (3)	C3—C4—C5—N1	−0.2 (3)
O4—Si1—O3—N2	−0.04 (12)	O4—C6—C7—C8	178.41 (18)
C11—Si1—O3—N2	88.43 (13)	N2—C6—C7—C8	−0.1 (3)
O2—Si1—O3—N2	−83.73 (12)	O4—N2'—C7—C8	178.41 (18)
C18—Si1—O3—N2	−173.18 (13)	C6'—N2'—C7—C8	−0.1 (3)
Si1—O1—N1—C1	2.8 (2)	C6—C7—C8—C9	0.1 (3)
Si1—O1—N1—C5	−176.97 (15)	N2'—C7—C8—C9	0.1 (3)
Si1—O2—C1—N1	−1.0 (2)	C7—C8—C9—C10	0.3 (3)
Si1—O2—C1—C2	179.15 (16)	O3—C6'—C10—C9	−178.11 (18)
O1—N1—C1—O2	−1.1 (2)	N2'—C6'—C10—C9	0.6 (3)
C5—N1—C1—O2	178.70 (17)	O3—N2—C10—C9	−178.11 (18)
O1—N1—C1—C2	178.76 (17)	C6—N2—C10—C9	0.6 (3)
C5—N1—C1—C2	−1.5 (3)	C8—C9—C10—C6'	−0.7 (3)
Si1—O3—N2—C6	0.1 (2)	C8—C9—C10—N2	−0.7 (3)
Si1—O3—N2—C10	178.91 (14)	C16—C11—C12—C13	−1.1 (3)
Si1—O4—C6—N2	0.1 (2)	Si1—C11—C12—C13	−178.69 (16)
Si1—O4—C6—C7	−178.56 (15)	C11—C12—C13—C14	0.7 (3)
O3—N2—C6—O4	−0.1 (2)	C12—C13—C14—C15	−0.3 (3)
C10—N2—C6—O4	−178.92 (17)	C12—C13—C14—C17	179.1 (2)
O3—N2—C6—C7	178.59 (16)	C13—C14—C15—C16	0.3 (3)
C10—N2—C6—C7	−0.2 (3)	C17—C14—C15—C16	−179.0 (2)
Si1—O2—N1'—C1'	−1.0 (2)	C14—C15—C16—C11	−0.8 (3)
Si1—O2—N1'—C2	179.15 (16)	C12—C11—C16—C15	1.1 (3)
Si1—O1—C1'—N1'	2.8 (2)	Si1—C11—C16—C15	178.77 (16)
Si1—O1—C1'—C5	−176.97 (15)	C23—C18—C19—C20	−2.3 (3)
O2—N1'—C1'—O1	−1.1 (2)	Si1—C18—C19—C20	178.54 (17)
C2—N1'—C1'—O1	178.76 (16)	C18—C19—C20—C21	−0.2 (3)
O2—N1'—C1'—C5	178.70 (17)	C19—C20—C21—C22	2.5 (3)
C2—N1'—C1'—C5	−1.5 (3)	C19—C20—C21—C24	−175.9 (2)
Si1—O4—N2'—C6'	0.1 (2)	C20—C21—C22—C23	−2.4 (3)
Si1—O4—N2'—C7	−178.56 (15)	C24—C21—C22—C23	176.0 (2)
Si1—O3—C6'—N2'	0.1 (2)	C19—C18—C23—C22	2.5 (3)
Si1—O3—C6'—C10	178.91 (14)	Si1—C18—C23—C22	−178.35 (16)
O4—N2'—C6'—O3	−0.1 (2)	C21—C22—C23—C18	−0.2 (3)

### Dimesitylbis[1-oxopyridin-2-olato(1-)]silicon(IV) (3) Crystal data

**Table:**

C_28_H_30_N_2_O_4_Si	*D*_x_ = 1.319 Mg m^−^^3^
*M**_r_* = 486.63	Cu *K*α radiation, λ = 1.54184 Å
Orthorhombic, *P*2_1_2_1_2_1_	Cell parameters from 11827 reflections
*a* = 12.5710 (2) Å	θ = 4.5–76.9°
*b* = 12.68898 (19) Å	µ = 1.15 mm^−^^1^
*c* = 15.3580 (2) Å	*T* = 100 K
*V* = 2449.80 (7) Å^3^	Block, colourless
*Z* = 4	0.09 × 0.07 × 0.06 mm
*F*(000) = 1032	

### Dimesitylbis[1-oxopyridin-2-olato(1-)]silicon(IV) (3) Data collection

**Table:**

XtaLAB Synergy, Dualflex, HyPix diffractometer	5138 independent reflections
Radiation source: micro-focus sealed X-ray tube, PhotonJet (Cu) X-ray Source	4847 reflections with *I* > 2σ(*I*)
Mirror monochromator	*R*_int_ = 0.048
Detector resolution: 10.0000 pixels mm^-1^	θ_max_ = 77.7°, θ_min_ = 4.5°
ω scans	*h* = −15→13
Absorption correction: multi-scan (CrysAlisPro; Rigaku OD, 2019)	*k* = −15→13
*T*_min_ = 0.674, *T*_max_ = 1.000	*l* = −19→19
22120 measured reflections	

### Dimesitylbis[1-oxopyridin-2-olato(1-)]silicon(IV) (3) Refinement

**Table:**

Refinement on *F*^2^	Secondary atom site location: difference Fourier map
Least-squares matrix: full	Hydrogen site location: inferred from neighbouring sites
*R*[*F*^2^ > 2σ(*F*^2^)] = 0.032	H-atom parameters constrained
*wR*(*F*^2^) = 0.079	*w* = 1/[σ^2^(*F*_o_^2^) + (0.0363*P*)^2^ + 0.5221*P*] where *P* = (*F*_o_^2^ + 2*F*_c_^2^)/3
*S* = 1.05	(Δ/σ)_max_ < 0.001
5138 reflections	Δρ_max_ = 0.27 e Å^−^^3^
324 parameters	Δρ_min_ = −0.25 e Å^−^^3^
0 restraints	Absolute structure: Flack *x* determined using 1985 quotients [(*I*^+^)-(*I*^-^)]/[(*I*^+^)+(*I*^-^)] (Parsons et al., 2013)
Primary atom site location: dual	Absolute structure parameter: −0.034 (17)

### Dimesitylbis[1-oxopyridin-2-olato(1-)]silicon(IV) (3) Special details

**Table:**

Geometry. All esds (except the esd in the dihedral angle between two l.s. planes) are estimated using the full covariance matrix. The cell esds are taken into account individually in the estimation of esds in distances, angles and torsion angles; correlations between esds in cell parameters are only used when they are defined by crystal symmetry. An approximate (isotropic) treatment of cell esds is used for estimating esds involving l.s. planes.
Refinement. Both bidentate ligands are disordered with the coplanar flips of themselves (0.68 (3):0.32 (3) and 0.61 (3):0.39 (3) for the rings containing C1/N1 and C6/N2, respectively). Due to resolution limitations, the disorder was modeled by refining the occupancies of the two atoms types (C and N) at the oxygen-coordinating portions of the rings. The occupancies at each site were constrained to sum to one and additionally sum to one C and one N atom between the two sites on each ring. The positional and anisotropic displacement parameters,respectively, at each site of disorder were constrained to be equivalent. It is understood that this type of disorder model will likely exhibit a weighted average of Si–O bond lengths, trending with the disorder ratios.

### Dimesitylbis[1-oxopyridin-2-olato(1-)]silicon(IV) (3) Fractional atomic coordinates and isotropic or equivalent isotropic displacement parameters (Å^2^)

**Table:**

	*x*	*y*	*z*	*U*_iso_*/*U*_eq_	Occ. (<1)
Si1	0.74087 (5)	0.70602 (4)	0.61168 (4)	0.01803 (13)	
O1	0.85697 (13)	0.71920 (12)	0.53026 (10)	0.0221 (3)	
O2	0.74861 (14)	0.56957 (11)	0.58288 (10)	0.0214 (3)	
O3	0.65878 (13)	0.71836 (12)	0.50381 (10)	0.0234 (3)	
O4	0.73653 (13)	0.84835 (11)	0.60520 (10)	0.0211 (3)	
N1	0.87289 (15)	0.62870 (15)	0.48904 (12)	0.0189 (4)	0.69 (3)
N2	0.65746 (17)	0.81659 (16)	0.47604 (13)	0.0220 (5)	0.62 (3)
C1	0.81438 (17)	0.54657 (16)	0.51912 (13)	0.0188 (5)	0.69 (3)
C2	0.8272 (2)	0.44746 (17)	0.48429 (15)	0.0231 (5)	
H2A	0.787040	0.389558	0.505675	0.028*	0.69 (3)
H2B	0.787040	0.389558	0.505675	0.028*	0.31 (3)
C3	0.8994 (2)	0.4339 (2)	0.41775 (17)	0.0285 (5)	
H3	0.909972	0.365897	0.393401	0.034*	
C4	0.9572 (2)	0.5195 (2)	0.38596 (16)	0.0282 (5)	
H4	1.006388	0.509933	0.339691	0.034*	
C5	0.94261 (18)	0.61737 (19)	0.42175 (15)	0.0228 (4)	
H5A	0.980582	0.676479	0.399952	0.027*	0.69 (3)
H5B	0.980582	0.676479	0.399952	0.027*	0.31 (3)
C6	0.70096 (17)	0.88806 (16)	0.53139 (14)	0.0207 (5)	0.62 (3)
C7	0.7032 (2)	0.99372 (18)	0.51074 (17)	0.0276 (5)	
H7A	0.735764	1.043126	0.548819	0.033*	0.62 (3)
H7B	0.735764	1.043126	0.548819	0.033*	0.38 (3)
C8	0.6575 (2)	1.0263 (2)	0.43398 (19)	0.0350 (6)	
H8	0.657349	1.098958	0.418960	0.042*	
C9	0.6110 (2)	0.9523 (2)	0.37793 (18)	0.0368 (6)	
H9	0.578834	0.974765	0.325137	0.044*	
C10	0.6121 (2)	0.8479 (2)	0.39921 (16)	0.0297 (5)	
H10	0.581545	0.797277	0.361044	0.036*	0.62 (3)
H10A	0.581545	0.797277	0.361044	0.036*	0.38 (3)
C11	0.60545 (17)	0.69383 (16)	0.67607 (13)	0.0181 (4)	
C12	0.58361 (19)	0.76268 (17)	0.74716 (14)	0.0205 (4)	
C13	0.48283 (19)	0.76687 (19)	0.78479 (14)	0.0234 (5)	
H13	0.471010	0.814780	0.831333	0.028*	
C14	0.39920 (19)	0.70421 (19)	0.75726 (15)	0.0253 (5)	
C15	0.42009 (19)	0.63418 (18)	0.68986 (16)	0.0240 (5)	
H15	0.364703	0.589014	0.670402	0.029*	
C16	0.51939 (18)	0.62777 (17)	0.64974 (14)	0.0211 (4)	
C17	0.6651 (2)	0.83405 (19)	0.79019 (15)	0.0250 (5)	
H17A	0.716867	0.791058	0.822061	0.038*	
H17B	0.629070	0.881774	0.830772	0.038*	
H17C	0.701983	0.875357	0.745519	0.038*	
C18	0.2900 (2)	0.7130 (2)	0.7976 (2)	0.0367 (6)	
H18A	0.296947	0.733539	0.858907	0.055*	
H18B	0.253707	0.644859	0.793814	0.055*	
H18C	0.248598	0.766439	0.766425	0.055*	
C19	0.5262 (2)	0.54616 (18)	0.57768 (16)	0.0258 (5)	
H19A	0.556781	0.578532	0.525422	0.039*	
H19B	0.454824	0.519629	0.564472	0.039*	
H19C	0.571523	0.487629	0.596638	0.039*	
C20	0.84221 (17)	0.70007 (16)	0.70814 (13)	0.0187 (4)	
C21	0.83877 (18)	0.61468 (17)	0.76842 (14)	0.0194 (4)	
C22	0.90158 (19)	0.61500 (17)	0.84337 (15)	0.0218 (4)	
H22	0.895115	0.558090	0.883212	0.026*	
C23	0.97317 (18)	0.69527 (18)	0.86195 (14)	0.0229 (4)	
C24	0.98178 (18)	0.77565 (19)	0.80091 (15)	0.0222 (4)	
H24	1.032215	0.830141	0.810637	0.027*	
C25	0.91924 (17)	0.77949 (18)	0.72584 (14)	0.0198 (4)	
C26	0.77207 (19)	0.51561 (17)	0.75647 (15)	0.0226 (5)	
H26A	0.699209	0.535362	0.740715	0.034*	
H26B	0.771184	0.475396	0.810947	0.034*	
H26C	0.802884	0.472274	0.710061	0.034*	
C27	1.0393 (2)	0.6943 (2)	0.94376 (16)	0.0335 (6)	
H27A	0.997851	0.723939	0.991945	0.050*	
H27B	1.103527	0.736728	0.934811	0.050*	
H27C	1.059530	0.621677	0.957731	0.050*	
C28	0.9418 (2)	0.87384 (18)	0.66723 (16)	0.0252 (5)	
H28A	0.934572	0.852709	0.606139	0.038*	
H28B	1.014281	0.899187	0.677740	0.038*	
H28C	0.890940	0.930276	0.680135	0.038*	
C1'	0.87289 (15)	0.62870 (15)	0.48904 (12)	0.0189 (4)	0.31 (3)
N1'	0.81438 (17)	0.54657 (16)	0.51912 (13)	0.0188 (5)	0.31 (3)
C6'	0.65746 (17)	0.81659 (16)	0.47604 (13)	0.0220 (5)	0.38 (3)
N2'	0.70096 (17)	0.88806 (16)	0.53139 (14)	0.0207 (5)	0.38 (3)

### Dimesitylbis[1-oxopyridin-2-olato(1-)]silicon(IV) (3) Atomic displacement parameters (Å^2^)

**Table:**

	*U*^11^	*U*^22^	*U*^33^	*U*^12^	*U*^13^	*U*^23^
Si1	0.0229 (3)	0.0143 (2)	0.0169 (3)	−0.0010 (2)	0.0020 (2)	−0.0006 (2)
O1	0.0290 (8)	0.0170 (7)	0.0204 (7)	−0.0031 (7)	0.0052 (6)	−0.0021 (6)
O2	0.0268 (8)	0.0173 (6)	0.0203 (7)	−0.0016 (6)	0.0064 (7)	−0.0020 (5)
O3	0.0298 (8)	0.0212 (7)	0.0193 (7)	−0.0037 (7)	−0.0015 (6)	0.0004 (6)
O4	0.0277 (8)	0.0169 (6)	0.0188 (7)	−0.0001 (6)	−0.0014 (7)	0.0020 (6)
N1	0.0214 (10)	0.0185 (9)	0.0169 (9)	−0.0006 (7)	−0.0006 (7)	0.0000 (7)
N2	0.0218 (10)	0.0240 (10)	0.0202 (9)	−0.0007 (8)	0.0016 (8)	0.0022 (8)
C1	0.0206 (10)	0.0207 (10)	0.0150 (9)	0.0002 (8)	−0.0008 (8)	0.0006 (8)
C2	0.0282 (12)	0.0185 (10)	0.0226 (10)	0.0010 (9)	−0.0038 (9)	−0.0003 (8)
C3	0.0327 (13)	0.0271 (11)	0.0256 (11)	0.0085 (10)	−0.0041 (10)	−0.0088 (9)
C4	0.0259 (12)	0.0384 (12)	0.0204 (11)	0.0056 (10)	0.0030 (10)	−0.0043 (10)
C5	0.0207 (10)	0.0312 (11)	0.0165 (10)	−0.0019 (9)	0.0002 (9)	0.0029 (9)
C6	0.0198 (10)	0.0216 (10)	0.0208 (10)	0.0005 (8)	0.0014 (8)	0.0033 (8)
C7	0.0265 (12)	0.0221 (11)	0.0341 (13)	0.0003 (9)	0.0049 (10)	0.0044 (10)
C8	0.0319 (13)	0.0321 (13)	0.0412 (14)	0.0058 (11)	0.0074 (12)	0.0170 (11)
C9	0.0332 (14)	0.0477 (15)	0.0295 (13)	0.0075 (12)	0.0003 (11)	0.0172 (12)
C10	0.0256 (12)	0.0430 (14)	0.0204 (11)	−0.0024 (10)	0.0001 (9)	0.0031 (10)
C11	0.0202 (10)	0.0169 (10)	0.0172 (9)	0.0027 (8)	−0.0003 (8)	0.0007 (8)
C12	0.0257 (11)	0.0185 (10)	0.0171 (10)	0.0051 (8)	−0.0025 (8)	0.0025 (8)
C13	0.0300 (12)	0.0222 (11)	0.0181 (10)	0.0072 (9)	0.0005 (9)	0.0005 (8)
C14	0.0241 (11)	0.0255 (11)	0.0261 (11)	0.0051 (10)	0.0037 (9)	0.0066 (9)
C15	0.0246 (11)	0.0213 (11)	0.0260 (12)	−0.0012 (9)	−0.0015 (9)	0.0030 (9)
C16	0.0251 (11)	0.0177 (10)	0.0204 (11)	−0.0004 (9)	−0.0004 (9)	0.0027 (8)
C17	0.0270 (12)	0.0266 (11)	0.0216 (10)	0.0020 (9)	0.0004 (9)	−0.0076 (9)
C18	0.0287 (12)	0.0378 (14)	0.0436 (15)	0.0028 (12)	0.0109 (11)	0.0019 (13)
C19	0.0280 (12)	0.0222 (11)	0.0273 (11)	−0.0060 (9)	0.0010 (10)	−0.0035 (9)
C20	0.0216 (10)	0.0158 (9)	0.0186 (9)	0.0015 (9)	0.0034 (8)	−0.0011 (8)
C21	0.0205 (10)	0.0166 (9)	0.0210 (10)	0.0026 (8)	0.0056 (8)	−0.0005 (8)
C22	0.0249 (11)	0.0182 (10)	0.0223 (11)	0.0044 (9)	0.0037 (9)	0.0023 (8)
C23	0.0235 (11)	0.0237 (11)	0.0215 (10)	0.0026 (9)	0.0029 (8)	0.0003 (9)
C24	0.0225 (11)	0.0202 (10)	0.0237 (10)	−0.0018 (9)	0.0035 (8)	−0.0030 (9)
C25	0.0220 (10)	0.0163 (10)	0.0211 (10)	0.0008 (8)	0.0040 (8)	−0.0007 (8)
C26	0.0276 (12)	0.0168 (10)	0.0232 (11)	−0.0015 (9)	0.0029 (9)	0.0027 (8)
C27	0.0375 (13)	0.0348 (13)	0.0281 (12)	−0.0056 (12)	−0.0054 (11)	0.0063 (11)
C28	0.0287 (12)	0.0208 (11)	0.0260 (12)	−0.0055 (9)	−0.0026 (10)	0.0025 (9)
C1'	0.0214 (10)	0.0185 (9)	0.0169 (9)	−0.0006 (7)	−0.0006 (7)	0.0000 (7)
N1'	0.0206 (10)	0.0207 (10)	0.0150 (9)	0.0002 (8)	−0.0008 (8)	0.0006 (8)
C6'	0.0218 (10)	0.0240 (10)	0.0202 (9)	−0.0007 (8)	0.0016 (8)	0.0022 (8)
N2'	0.0198 (10)	0.0216 (10)	0.0208 (10)	0.0005 (8)	0.0014 (8)	0.0033 (8)

### Dimesitylbis[1-oxopyridin-2-olato(1-)]silicon(IV) (3) Geometric parameters (Å, º)

**Table:**

Si1—O1	1.9291 (16)	C11—C12	1.425 (3)
Si1—O2	1.7896 (15)	C11—C16	1.427 (3)
Si1—O3	1.9581 (16)	C12—C13	1.394 (3)
Si1—O4	1.8096 (15)	C12—C17	1.519 (3)
Si1—C11	1.975 (2)	C13—H13	0.9500
Si1—C20	1.955 (2)	C13—C14	1.384 (4)
O1—N1	1.326 (2)	C14—C15	1.389 (4)
O1—C1'	1.326 (2)	C14—C18	1.510 (3)
O2—C1	1.314 (3)	C15—H15	0.9500
O2—N1'	1.314 (3)	C15—C16	1.395 (3)
O3—N2	1.317 (3)	C16—C19	1.518 (3)
O3—C6'	1.317 (3)	C17—H17A	0.9800
O4—C6	1.319 (3)	C17—H17B	0.9800
O4—N2'	1.319 (3)	C17—H17C	0.9800
N1—C1	1.357 (3)	C18—H18A	0.9800
N1—C5	1.363 (3)	C18—H18B	0.9800
N2—C6	1.358 (3)	C18—H18C	0.9800
N2—C10	1.369 (3)	C19—H19A	0.9800
C1—C2	1.376 (3)	C19—H19B	0.9800
C2—H2A	0.9500	C19—H19C	0.9800
C2—H2B	0.9500	C20—C21	1.426 (3)
C2—C3	1.378 (4)	C20—C25	1.424 (3)
C2—N1'	1.376 (3)	C21—C22	1.396 (3)
C3—H3	0.9500	C21—C26	1.522 (3)
C3—C4	1.394 (4)	C22—H22	0.9500
C4—H4	0.9500	C22—C23	1.389 (3)
C4—C5	1.371 (3)	C23—C24	1.390 (3)
C5—H5A	0.9500	C23—C27	1.507 (3)
C5—H5B	0.9500	C24—H24	0.9500
C5—C1'	1.363 (3)	C24—C25	1.396 (3)
C6—C7	1.378 (3)	C25—C28	1.524 (3)
C7—H7A	0.9500	C26—H26A	0.9800
C7—H7B	0.9500	C26—H26B	0.9800
C7—C8	1.375 (4)	C26—H26C	0.9800
C7—N2'	1.378 (3)	C27—H27A	0.9800
C8—H8	0.9500	C27—H27B	0.9800
C8—C9	1.402 (4)	C27—H27C	0.9800
C9—H9	0.9500	C28—H28A	0.9800
C9—C10	1.365 (4)	C28—H28B	0.9800
C10—H10	0.9500	C28—H28C	0.9800
C10—H10A	0.9500	C1'—N1'	1.357 (3)
C10—C6'	1.369 (3)	C6'—N2'	1.358 (3)
			
O1—Si1—O3	80.99 (7)	C14—C13—C12	122.8 (2)
O1—Si1—C11	169.59 (8)	C14—C13—H13	118.6
O1—Si1—C20	90.09 (8)	C13—C14—C15	116.8 (2)
O2—Si1—O1	83.25 (7)	C13—C14—C18	121.5 (2)
O2—Si1—O3	84.09 (7)	C15—C14—C18	121.7 (2)
O2—Si1—O4	162.48 (8)	C14—C15—H15	118.8
O2—Si1—C11	95.44 (8)	C14—C15—C16	122.4 (2)
O2—Si1—C20	96.57 (8)	C16—C15—H15	118.8
O3—Si1—C11	88.61 (8)	C11—C16—C19	124.4 (2)
O4—Si1—O1	84.30 (7)	C15—C16—C11	121.3 (2)
O4—Si1—O3	81.82 (7)	C15—C16—C19	114.4 (2)
O4—Si1—C11	94.57 (8)	C12—C17—H17A	109.5
O4—Si1—C20	95.73 (8)	C12—C17—H17B	109.5
C20—Si1—O3	170.93 (8)	C12—C17—H17C	109.5
C20—Si1—C11	100.32 (9)	H17A—C17—H17B	109.5
N1—O1—Si1	110.40 (13)	H17A—C17—H17C	109.5
C1'—O1—Si1	110.40 (13)	H17B—C17—H17C	109.5
C1—O2—Si1	115.67 (13)	C14—C18—H18A	109.5
N1'—O2—Si1	115.67 (13)	C14—C18—H18B	109.5
N2—O3—Si1	110.86 (13)	C14—C18—H18C	109.5
C6'—O3—Si1	110.86 (13)	H18A—C18—H18B	109.5
C6—O4—Si1	116.03 (13)	H18A—C18—H18C	109.5
N2'—O4—Si1	116.03 (13)	H18B—C18—H18C	109.5
O1—N1—C1	114.88 (18)	C16—C19—H19A	109.5
O1—N1—C5	123.39 (19)	C16—C19—H19B	109.5
C1—N1—C5	121.73 (19)	C16—C19—H19C	109.5
O3—N2—C6	115.09 (18)	H19A—C19—H19B	109.5
O3—N2—C10	123.9 (2)	H19A—C19—H19C	109.5
C6—N2—C10	120.9 (2)	H19B—C19—H19C	109.5
O2—C1—N1	115.10 (18)	C21—C20—Si1	120.11 (16)
O2—C1—C2	124.5 (2)	C25—C20—Si1	124.08 (16)
N1—C1—C2	120.4 (2)	C25—C20—C21	115.75 (19)
C1—C2—H2A	120.7	C20—C21—C26	124.5 (2)
C1—C2—C3	118.6 (2)	C22—C21—C20	121.1 (2)
C3—C2—H2A	120.7	C22—C21—C26	114.42 (19)
C3—C2—H2B	120.7	C21—C22—H22	118.7
N1'—C2—H2B	120.7	C23—C22—C21	122.6 (2)
N1'—C2—C3	118.6 (2)	C23—C22—H22	118.7
C2—C3—H3	119.8	C22—C23—C24	116.8 (2)
C2—C3—C4	120.4 (2)	C22—C23—C27	121.5 (2)
C4—C3—H3	119.8	C24—C23—C27	121.7 (2)
C3—C4—H4	120.1	C23—C24—H24	118.7
C5—C4—C3	119.7 (2)	C23—C24—C25	122.6 (2)
C5—C4—H4	120.1	C25—C24—H24	118.7
N1—C5—C4	119.0 (2)	C20—C25—C28	124.7 (2)
N1—C5—H5A	120.5	C24—C25—C20	121.1 (2)
C4—C5—H5A	120.5	C24—C25—C28	114.2 (2)
C4—C5—H5B	120.5	C21—C26—H26A	109.5
C1'—C5—C4	119.0 (2)	C21—C26—H26B	109.5
C1'—C5—H5B	120.5	C21—C26—H26C	109.5
O4—C6—N2	114.80 (18)	H26A—C26—H26B	109.5
O4—C6—C7	124.2 (2)	H26A—C26—H26C	109.5
N2—C6—C7	120.9 (2)	H26B—C26—H26C	109.5
C6—C7—H7A	120.6	C23—C27—H27A	109.5
C8—C7—C6	118.8 (2)	C23—C27—H27B	109.5
C8—C7—H7A	120.6	C23—C27—H27C	109.5
C8—C7—H7B	120.6	H27A—C27—H27B	109.5
C8—C7—N2'	118.8 (2)	H27A—C27—H27C	109.5
N2'—C7—H7B	120.6	H27B—C27—H27C	109.5
C7—C8—H8	120.0	C25—C28—H28A	109.5
C7—C8—C9	119.9 (2)	C25—C28—H28B	109.5
C9—C8—H8	120.0	C25—C28—H28C	109.5
C8—C9—H9	120.0	H28A—C28—H28B	109.5
C10—C9—C8	120.0 (2)	H28A—C28—H28C	109.5
C10—C9—H9	120.0	H28B—C28—H28C	109.5
N2—C10—H10	120.3	O1—C1'—C5	123.39 (19)
C9—C10—N2	119.4 (2)	O1—C1'—N1'	114.88 (18)
C9—C10—H10	120.3	N1'—C1'—C5	121.73 (19)
C9—C10—H10A	120.3	O2—N1'—C2	124.5 (2)
C9—C10—C6'	119.4 (2)	O2—N1'—C1'	115.10 (18)
C6'—C10—H10A	120.3	C1'—N1'—C2	120.4 (2)
C12—C11—Si1	120.12 (16)	O3—C6'—C10	123.9 (2)
C12—C11—C16	115.5 (2)	O3—C6'—N2'	115.09 (18)
C16—C11—Si1	123.89 (16)	N2'—C6'—C10	120.9 (2)
C11—C12—C17	124.7 (2)	O4—N2'—C7	124.2 (2)
C13—C12—C11	121.1 (2)	O4—N2'—C6'	114.80 (18)
C13—C12—C17	114.21 (19)	C6'—N2'—C7	120.9 (2)
C12—C13—H13	118.6		
			
Si1—O1—N1—C1	−7.0 (2)	C3—C4—C5—N1	1.1 (4)
Si1—O1—N1—C5	173.30 (17)	C3—C4—C5—C1'	1.1 (4)
Si1—O1—C1'—C5	173.30 (17)	C4—C5—C1'—O1	177.0 (2)
Si1—O1—C1'—N1'	−7.0 (2)	C4—C5—C1'—N1'	−2.8 (3)
Si1—O2—C1—N1	4.9 (2)	C5—N1—C1—O2	−178.44 (19)
Si1—O2—C1—C2	−176.23 (18)	C5—N1—C1—C2	2.6 (3)
Si1—O2—N1'—C2	−176.23 (18)	C5—C1'—N1'—O2	−178.44 (19)
Si1—O2—N1'—C1'	4.9 (2)	C5—C1'—N1'—C2	2.6 (3)
Si1—O3—N2—C6	−7.2 (2)	C6—N2—C10—C9	0.3 (4)
Si1—O3—N2—C10	175.59 (18)	C6—C7—C8—C9	−0.9 (4)
Si1—O3—C6'—C10	175.59 (18)	C7—C8—C9—C10	−0.6 (4)
Si1—O3—C6'—N2'	−7.2 (2)	C8—C7—N2'—O4	−175.9 (2)
Si1—O4—C6—N2	9.7 (2)	C8—C7—N2'—C6'	2.0 (4)
Si1—O4—C6—C7	−172.30 (18)	C8—C9—C10—N2	0.9 (4)
Si1—O4—N2'—C7	−172.30 (18)	C8—C9—C10—C6'	0.9 (4)
Si1—O4—N2'—C6'	9.7 (2)	C9—C10—C6'—O3	177.3 (2)
Si1—C11—C12—C13	169.91 (16)	C9—C10—C6'—N2'	0.3 (4)
Si1—C11—C12—C17	−12.0 (3)	C10—N2—C6—O4	176.3 (2)
Si1—C11—C16—C15	−170.09 (17)	C10—N2—C6—C7	−1.8 (3)
Si1—C11—C16—C19	9.9 (3)	C10—C6'—N2'—O4	176.3 (2)
Si1—C20—C21—C22	−172.32 (16)	C10—C6'—N2'—C7	−1.8 (3)
Si1—C20—C21—C26	10.1 (3)	C11—Si1—O2—C1	162.73 (15)
Si1—C20—C25—C24	173.31 (16)	C11—Si1—O2—N1'	162.73 (15)
Si1—C20—C25—C28	−6.9 (3)	C11—Si1—O4—C6	−98.55 (16)
O1—Si1—O2—C1	−6.88 (15)	C11—Si1—O4—N2'	−98.55 (16)
O1—Si1—O2—N1'	−6.88 (15)	C11—C12—C13—C14	1.2 (3)
O1—Si1—O4—C6	71.06 (15)	C12—C11—C16—C15	2.2 (3)
O1—Si1—O4—N2'	71.06 (15)	C12—C11—C16—C19	−177.9 (2)
O1—N1—C1—O2	1.8 (3)	C12—C13—C14—C15	0.9 (3)
O1—N1—C1—C2	−177.11 (19)	C12—C13—C14—C18	−178.1 (2)
O1—N1—C5—C4	177.0 (2)	C13—C14—C15—C16	−1.4 (3)
O1—C1'—N1'—O2	1.8 (3)	C14—C15—C16—C11	−0.2 (3)
O1—C1'—N1'—C2	−177.11 (19)	C14—C15—C16—C19	179.8 (2)
O2—Si1—O4—C6	26.2 (4)	C16—C11—C12—C13	−2.7 (3)
O2—Si1—O4—N2'	26.2 (4)	C16—C11—C12—C17	175.4 (2)
O2—C1—C2—C3	−179.6 (2)	C17—C12—C13—C14	−177.1 (2)
O3—Si1—O2—C1	74.71 (15)	C18—C14—C15—C16	177.6 (2)
O3—Si1—O2—N1'	74.71 (15)	C20—Si1—O2—C1	−96.19 (16)
O3—Si1—O4—C6	−10.62 (15)	C20—Si1—O2—N1'	−96.19 (16)
O3—Si1—O4—N2'	−10.62 (15)	C20—Si1—O4—C6	160.58 (15)
O3—N2—C6—O4	−1.0 (3)	C20—Si1—O4—N2'	160.58 (15)
O3—N2—C6—C7	−179.0 (2)	C20—C21—C22—C23	−2.5 (3)
O3—N2—C10—C9	177.3 (2)	C21—C20—C25—C24	−3.9 (3)
O3—C6'—N2'—O4	−1.0 (3)	C21—C20—C25—C28	175.9 (2)
O3—C6'—N2'—C7	−179.0 (2)	C21—C22—C23—C24	−1.4 (3)
O4—Si1—O2—C1	38.1 (4)	C21—C22—C23—C27	179.4 (2)
O4—Si1—O2—N1'	38.1 (4)	C22—C23—C24—C25	2.6 (3)
O4—C6—C7—C8	−175.9 (2)	C23—C24—C25—C20	0.2 (3)
N1—C1—C2—C3	−0.8 (3)	C23—C24—C25—C28	−179.6 (2)
N2—C6—C7—C8	2.0 (4)	C25—C20—C21—C22	5.0 (3)
C1—N1—C5—C4	−2.8 (3)	C25—C20—C21—C26	−172.6 (2)
C1—C2—C3—C4	−0.8 (4)	C26—C21—C22—C23	175.3 (2)
C2—C3—C4—C5	0.6 (4)	C27—C23—C24—C25	−178.2 (2)
C3—C2—N1'—O2	−179.6 (2)	N1'—C2—C3—C4	−0.8 (4)
C3—C2—N1'—C1'	−0.8 (3)	N2'—C7—C8—C9	−0.9 (4)
